# Supplementary material for: Thresholds for clinical practice that directly link handgrip strength to remaining years of life: estimates based on longitudinal observational data
Source: BMJ Open. 2022 Jul 22;12(7):e058489. doi: 10.1136/bmjopen-2021-058489 (PMC9315893; doi:10.1136/bmjopen-2021-058489)
Supplement: Supplementary data [file bmjopen-2021-058489supp001.pdf]

SUPPLEMENTARY MATERIAL

Initial sample

N = 12,137 (6,931 women; 5,206 men)

↓

Sample after considering demographic characteristics, anthropometric traits, and HGS

Keeping individuals aged 50 to 80;
keeping Caucasian individuals;
excluding male outliers with height < 150 cm or height > 210 cm;
excluding female outliers with height < 130 cm or height > 190 cm;
excluding outliers with weight < 30 kg or weight > 150 kg;
excluding outliers with handgrip strength < 6 kg or handgrip strength > 80 kg

N = 8,309 (4,668 women; 3,641 men)

↓

Sample after dropping illogical exposure

Excluding individuals that supposedly died before the survey took place

N = 8,306 (4,666 women; 3,640 men)

↓

Final sample after excluding observations with residuals larger than 2.5 SD

N = 8,156 (4,573 women; 3,583 men)

Figure A1: Sample construction

Scherbov S, *et al. BMJ Open* 2022; 12:e058489. doi: 10.1136/bmjopen-2021-058489

	**Model 1 (without education)**	**Model 2 (with education)**
**Men**		
Reference: [0.0 SD, 0.5 SD)	1.00 (reference)	1.00 (reference)
Strong: [0.5 SD, 3.0 SD)	0.93 (0.69-1.25)	0.93 (0.69-1.26)
Weak 1: [-0.5 SD, 0.0 SD)	1.67 (1.23-2.26)	1.68 (1.24-2.27)
Weak 2: [-1.0 SD, -0.5 SD)	2.02 (1.49-2.75)	1.98 (1.46-2.69)
Weak 3: [-2.0 SD, -1.0 SD)	2.40 (1.77-3.26)	2.39 (1.76-3.25)
Weak 4: [-3.0 SD, -2.0 SD)	2.34 (1.40-3.93)	2.32 (1.39-3.89)
Age in years	1.09 (1.07-1.10)	1.09 (1.07-1.10)
Years of education		0.96 (0.93-0.98)
Max log likelihood	-2358.9	-2353.0
LR test statistic	244.6	256.4
Total time at risk	21864	21864
Events	529	529
**Women**		
Reference: [0.0 SD, 0.5 SD)	1.00 (reference)	1.00 (reference)
Strong: [0.5 SD, 3.0 SD)	0.90 (0.66-1.22)	0.89 (0.66-1.22)
Weak 1: [-0.5 SD, 0.0 SD)	1.32 (0.96-1.82)	1.30 (0.94-1.79)
Weak 2: [-1.0 SD, -0.5 SD)	1.65 (1.20-2.28)	1.62 (1.18-2.24)
Weak 3: [-2.0 SD, -1.0 SD)	1.85 (1.34-2.55)	1.83 (1.32-2.53)
Weak 4: [-3.0 SD, -2.0 SD)	3.03 (1.83-5.04)	2.98 (1.79-4.96)
Age in years	1.10 (1.08-1.12)	1.10 (1.08-1.11)
Years of education		0.93 (0.90-0.96)
Max log likelihood	-2192.8	-2181.7
LR test statistic	212.6	234.8
Total time at risk	28500	28500
Events	449	449

95% CI in parentheses, years of education < 20 years

Table A1: Hazard ratios for mortality by gender and st_HGS group, with and without years of education as control

Scherbov S, *et al. BMJ Open* 2022; 12:e058489. doi: 10.1136/bmjopen-2021-058489

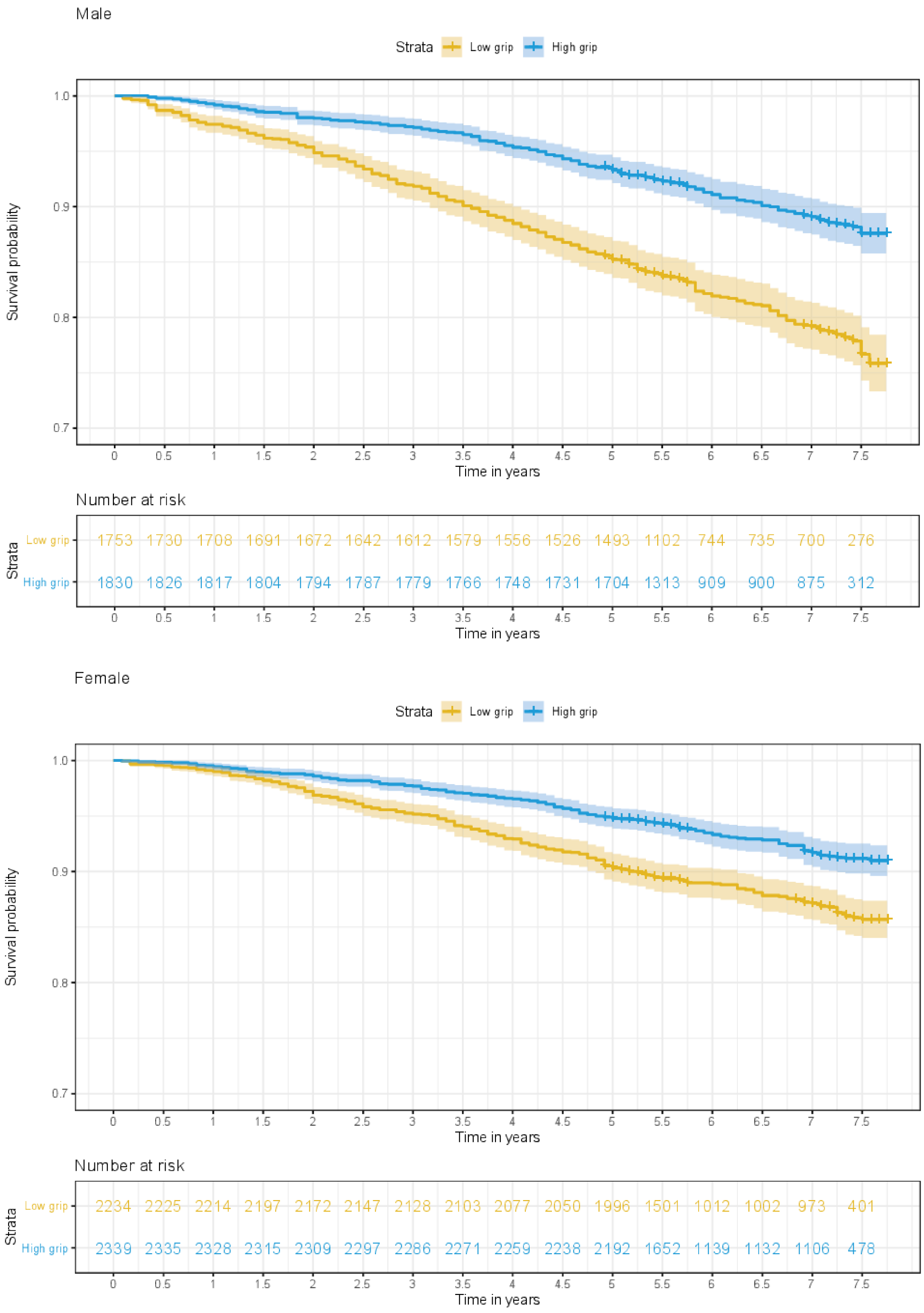

Figure A2: Survival and numbers at risk, by gender and standardised handgrip strength

“High grip” refers to the strong and reference group and “low grip” to groups Weak1-4
