# Supplementary material for: Thresholds for clinical practice that directly link handgrip strength to remaining years of life: estimates based on longitudinal observational data
Source: BMJ Open. 2022 Jul 22;12(7):e058489. doi: 10.1136/bmjopen-2021-058489 (PMC9315893; doi:10.1136/bmjopen-2021-058489)
Supplement: Author's manuscript [file bmjopen-2021-058489.draft_revisions.pdf]

BMJ Open

BMJ Open is committed to open peer review. As part of this commitment we make the peer review history of every article we publish publicly available.

When an article is published we post the peer reviewers' comments and the authors' responses online. We also post the versions of the paper that were used during peer review. These are the versions that the peer review comments apply to.

The versions of the paper that follow are the versions that were submitted during the peer review process. They are not the versions of record or the final published versions. They should not be cited or distributed as the published version of this manuscript.

BMJ Open is an open access journal and the full, final, typeset and author-corrected version of record of the manuscript is available on our site with no access controls, subscription charges or pay-per-view fees (http://bmjopen.bmj.com).

If you have any questions on BMJ Open's open peer review process please email info.bmjopen@bmj.com

BMJ Open

Thresholds for clinical practice that directly link handgrip strength to remaining years of life: estimates based on longitudinal observational data

Journal:	*BMJ Open*
Manuscript ID	bmjopen-2021-058489
Article Type:	Original research
Date Submitted by the Author:	05-Nov-2021

Complete List of Authors:	Scherbov, Sergei; International Institute for Applied Systems Analysis<br>Spitzer, Sonja; University of Vienna, Department of Demography<br>Steiber, Nadia; University of Vienna, Department of Sociology; Institute for Advanced Studies, Department of Sociology

Keywords:	GERIATRIC MEDICINE, PUBLIC HEALTH, STATISTICS & RESEARCH METHODS

SCHOLARONE™
Manuscripts

I, the Submitting Author has the right to grant and does grant on behalf of all authors of the Work (as defined in the below author licence), an exclusive licence and/or a non-exclusive licence for contributions from authors who are: i) UK Crown employees; ii) where BMJ has agreed a CC-BY licence shall apply, and/or iii) in accordance with the terms applicable for US Federal Government officers or employees acting as part of their official duties; on a worldwide, perpetual, irrevocable, royalty-free basis to BMJ Publishing Group Ltd ("BMJ") its licensees and where the relevant Journal is co-owned by BMJ to the co-owners of the Journal, to publish the Work in this journal and any other BMJ products and to exploit all rights, as set out in our licence.

The Submitting Author accepts and understands that any supply made under these terms is made by BMJ to the Submitting Author unless you are acting as an employee on behalf of your employer or a postgraduate student of an affiliated institution which is paying any applicable article publishing charge ("APC") for Open Access articles. Where the Submitting Author wishes to make the Work available on an Open Access basis (and intends to pay the relevant APC), the terms of reuse of such Open Access shall be governed by a Creative Commons licence – details of these licences and which Creative Commons licence will apply to this Work are set out in our licence referred to above.

Other than as permitted in any relevant BMJ Author's Self Archiving Policies, I confirm this Work has not been accepted for publication elsewhere, is not being considered for publication elsewhere and does not duplicate material already published. I confirm all authors consent to publication of this Work and authorise the granting of this licence.

For peer review only

TITLE PAGE

Thresholds for clinical practice that directly link handgrip strength to remaining years of life: estimates based on longitudinal observational data

Dr. Sergei Scherbov

Principal Research Scholar, Population and Just Societies Program, International Institute for Applied Systems Analysis (IIASA), Wittgenstein Centre for Demography and Global Human Capital (IIASA, OeAW, University of Vienna), Schlossplatz 1, 2361 Laxenburg, Austria

E-mail: scherbov@iiasa.ac.at

Sonja Spitzer, Ph.D.

Postdoctoral Researcher, Department of Demography, University of Vienna, Wittgenstein Centre for Demography and Global Human Capital (IIASA, OeAW, University of Vienna), Vordere Zollamtsstraße 3, 1030 Vienna, Austria

E-mail: sonja.spitzer@univie.ac.at

Dr. Nadia Steiber (corresponding author)

Full Professor, Department of Sociology, University of Vienna, Rooseveltplatz 2, 1090 Vienna, Austria and Institute for Advanced Studies, Vienna, Austria

E-mail: nadia.steiber@univie.ac.at

Phone: +43 1 4277 49290

AUTHOR CONTRIBUTION

SSC and NS designed the study, SSC was responsible for data curation and the statistical analyses, NS wrote the original draft, and SSP was responsible for reviewing and editing the manuscript as well as data visualisation. All authors have verified the underlying data and are responsible for the overall content. The corresponding author attests that all listed authors meet authorship criteria and that no others meeting the criteria have been omitted.

PATIENT CONSENT

Not applicable

DECLARATION OF INTEREST

We declare no competing interests

WORD COUNT: 3,838

For peer review only - http://bmjopen.bmj.com/site/about/guidelines.xhtml

ABSTRACT

Objective: Muscle strength is a powerful predictor of mortality that can quickly and inexpensively be assessed via handgrip strength (HGS) measures. What is missing for clinical practice are empirically meaningful cut-off points that apply to the general population and consider the correlation of HGS with gender and body height, and its decline during processes of *normal* ageing. This study provides standardised thresholds that directly link HGS to remaining life expectancy (RLE) and thus enable practitioners to detect patients with an increased mortality risk early on.

Design: Relying on representative observational data from the Health and Retirement Study, HGS of survey participants was z-standardised by gender, age, and body height. We defined six HGS groups based on cut-off points in standard deviation (SD), use them as predictors in survival analyses with a 9-year follow-up, and provide RLE by gender based on a Gompertz model for each HGS group.

Participants: 8,156 US-American women and men aged 50 to 80

Main outcome measures: z-standardised HGS and all-cause mortality

Results: Already slight negative deviations in HGS from the reference group with [0·0 SD, 0·5 SD) have substantial effects on survival. RLE among 60-year-olds with standardised HGS of [-0·5 SD, 0·0 SD) is 3·0 / 1·4 years lower for men / women than that of the reference group, increasing to a difference of 4·1 / 2·6 years in the group with HGS of [-1·0 SD, -0·5 SD). By contrast, we find no benefit of strong HGS related to survival.

Conclusions: HGS varies substantially with gender, age, and body height, confirming that it is of utmost importance to consider these heterogeneities when defining reference groups and risk thresholds. Moreover, survival appears to decrease at much higher levels of muscle strength than assumed in the previous literature, suggesting that practitioners should already start being concerned when HGS is slightly below that of the reference group.

For peer review only

For peer review only - http://bmjopen.bmj.com/site/about/guidelines.xhtml

Strengths and limitations of this study:

- We develop a clinical definition of low handgrip strength related to increased mortality risk, and provide cut-off points along with straight-forward visualisations to be used in medical practice.
- In addition to hazard ratios, we present, for the first time, estimates of remaining life expectancy related to the cut-off points, enabling a much more intuitive interpretation of mortality risk that can easily be communicated to patients.
- Our evidence points towards a threshold value for defining a critically weak handgrip that is much higher compared to what has been assumed in gerontological research so far. This suggests that routine measurement of HGS in medical practice can be used for a much earlier detection of individuals who are at risk of premature mortality.
- Previous work suggests substantial differences in reference handgrip strength by race Due to small numbers of observations from other races, this study provides cut-offs based on data from Caucasian individuals only.

For peer review only

INTRODUCTION

Muscle strength, as frequently measured by handgrip strength (HGS), is a well-established indicator of functional status that has been widely used as a key component of frailty phenotypes[1,2] as well as to diagnose sarcopenia.[3,4] Going beyond the use of HGS measurement in gerontological assessments, a large and growing body of research is concerned with its prognostic value for future health conditions,[5] showing that HGS is a powerful predictor of adverse health outcomes such as disability, cognitive decline, and eventually mortality.[6–8]

Given its strong association with the functional status of older individuals and its high prognostic value for future morbidity and mortality, HGS has been promoted as a biomarker of healthy ageing, arguing that it is a better predictor of survival and the maintenance of good health than chronological age.[9] The measurement of HGS has been suggested as a screening tool for the vulnerability of older individuals that is much simpler and cost-effective compared to comprehensive geriatric assessments.[10,11]

Whereas there is consensus that HGS could be routinely used to screen for those whose future health is at risk, and who may require intervention, research on empirically relevant threshold values to be used in such screenings is scarce.[12] Recent studies on the predictive value of HGS for mortality have either used a continuous measure of HGS in kilograms (kg), or broad and arbitrary classifications of HGS into quantiles.[6,12–15] Moreover, studies are often based on small, non-representative samples that are targeted

For peer review only - http://bmjopen.bmj.com/site/about/guidelines.xhtml

at patients suffering from certain diseases only[16,17], or use mediating health outcomes to define thresholds. The Foundation of the National Institutes of Health,[18] for example, constructed cut-off points in terms of kg based on the statistical associations between HGS and low gait speed, which were used as mortality predictors in a subsequent study.[19]

The most popular threshold for weak HGS – by now also frequently used to predict survival – is not actually conceptualised with mortality in mind, but for the diagnosis of sarcopenia, instead. In 2010, the European Working Group on Sarcopenia in Older Persons (EWGSOP) recommended a cut-off at two standard deviations (SD) below the mean value of HGS taken from a normative, healthy reference population to define muscle weakness.[3] In 2019, the group has updated their thresholds, now suggesting a cut-off at 2·5 SD to define low HGS, translating to < 27 kg for men and < 16 kg for women.[4] This simple suggestion has become a common criterion, used worldwide, for the diagnosis of sarcopenia in geriatric assessment and gerontological research. Moreover, it is used in numerous studies as a threshold to predict mortality differentials.

However, when defining thresholds as two or 2·5 SD below the mean value of a healthy reference population, i.e. t-scores, the decline of HGS during processes of *normal* ageing from age 30 to 40 onwards[21] – associated with levels of physical activity, anabolic responsiveness to protein intake, and hormonal status[23] – cannot be accounted for. This decline is not indicative of pathological ageing, i.e. not associated with mortality risks above those seen in other individuals of the same chronological age. Using a threshold based on t-scores would imply that the majority of older individuals is at a higher risk of dying, simply reflecting lower life expectancy of the old rather than providing an effective tool to detect the most vulnerable within each age group. Moreover, HGS increases substantially with body height,[21] which is neither an indicator of absent sarcopenia nor of higher survival. Adjusting HGS for body height thus has the potential to improve mortality risk assessments.[14] In a similar vein, women have a lower average HGS than men,[22] and HGS varies by race.[20]

In their most recent report, EWGSOP thus highlight the need for studies establishing gender- and region-specific threshold values to improve outcome prediction.[4] Going one step further and accounting for processes of *normal* ageing as well as the associated of HGS with anthropometric traits, we propose standardised threshold values that consider the inherent, non-informative variations in muscle strength with gender, age, and body height, i.e. z-scores, using separate reference categories for each gender-age-height group. These enable practitioners to clearly detect patients that deviate from the norm, i.e. their reference group, and thus have a higher, or potentially lower, risk of death. Moreover, age-standardised thresholds are feasible even when data for the entire age distribution are not available.

For peer review only - http://bmjopen.bmj.com/site/about/guidelines.xhtml

The aim of the present study is thus to define standardised threshold values for HGS that are associated with a substantially increasing risk of mortality, using z-scores. We provide estimates based on representative data for US-Americans aged 50 and above, irrespective of their health status, and directly link muscle strength to mortality, without any deviations to mediating diseases. I addition to defining standardised thresholds, we contribute to previous work by providing estimates of remaining life expectancy (RLE) associated with each of the cut-off points. In contrast to hazard ratios (HR), RLE informs about actual life expectancy differences between HGS groups and can be communicated to patients more intuitively – a feature that was hitherto ignored in the literature. Moreover, we produce clear illustrations of the relationship between tested HGS and the risk groups, which can routinely be employed in medical practice and used as a low-entry starting point for further geriatric assessments and health-enhancing patient interventions.

METHODS

Data, sample, and study design

Our analysis is based on the Health and Retirement Study (HRS), a longitudinal panel survey providing representative data for the US-American population aged 50 and older.[24] In 2006, half of the survey participants were randomly selected to perform a HGS test, with the other half completing the test in 2008. HRS can be linked with mortality register data from the National Death Index, which allowed us to follow survey participants until December 2014.

We restrict our sample to individuals aged 50 to 80, keeping all observations with HGS of at least five kg. To remove outliers in terms of anthropometric traits, we only keep women with a body height of 130 to 190 cm and men with at least 150 cm. We focus on Caucasian individuals, because previous work shows a need for separate HGS thresholds by race,[20] yet the number of observations for the other ethnic groups was too small for heterogeneity analyses. Finally, we drop cases with residuals larger than 2·5 SD from gender-specific linear regressions of age and body height on HGS (next subsection for details). This leaves us with a sample of 8,156 individuals to predict death with a follow-up period of up to 9 years, during which 978 participants died. More detailed sample characteristics are provided in Table 1.

	Men	Women
Grip strength, kg, mean (SD)	42·1 (8·4)	25·5 (5·5)
Death during follow-up	529 (14·8%)	449 (9·8%)
Age, years, median (IQR)	68·2 (61·8-73·3)	67·7 (61·6-73·1)
Height, cm, median (IQR)	174·6 (170·2-179·1)	160·0 (156·2-164·5)
N	3,583	4,573

SD = standard deviation; IQR = interquartile range 75%-25%

Table 1: Participant characteristics stratified by gender

For peer review only - http://bmjopen.bmj.com/site/about/guidelines.xhtml

Handgrip strength measurement and z-standardisation by gender, age, and body height

HGS was measured using a Smedley spring-type hand dynamometer. Two measures were taken from each hand. Following published recommendations,[25] we use the maximum value achieved with either hand for our analyses.

As mentioned above, there is strong variation in HGS by gender, age, and body height that is not directly linked to differences in mortality risks. We thus use a straight-forward regression technique to adjust HGS values for these characteristics. First, we regress HGS on age and body height, using separate linear models for women and men. Squared age and height terms were not significant. Regression analyses confirm the strong differences in HGS by gender, age, and body height. More specifically, the expected HGS at baseline in 2006/2008 at ages 50 to 80 can be expressed as:

Men: $HGS_m = 7.23 + 0.38 \times height(cm) - 0.46 \times age(yr)$ (Adjusted R2: 0·28)

Women: $HGS_f = 6.00 + 0.25 \times height(cm) - 0.30 \times age(yr)$ (Adjusted R2: 0·27)

Second, we calculate the standardised residuals from the above equations, applying a z-standardisation with mean of 0 and SD of 1 to obtain a measure of HGS for each observation that is standardised for gender, age, and body height, in the following denoted as st_HGS_i. This allows for the construction of reference categories for each gender-age-height-combination. Deviations in st_HGS_i from these reference categories enable the detection of mortality risk differentials.

In a third step we define thresholds that allow for an early detection of patients with increased mortality risk. Attempts at defining optimal cut-off points using a log-rank method or similar methods rendered non-robust results due to the monotonic increase in the mortality risk with st_HGS_i. We thus construct six groups based on SD thresholds, as shown in Table 2, Rows 1 and 2. Given the high prevalence of chronic diseases in the study population, which is aged 50 and above, we defined the 'healthy' reference group as those with st_HGS_i at or slightly above the mean [0·0 SD, 0·5 SD). The comparison groups comprise those up to 0·5 SD below the mean (Weak 1), those with less than a half to a full SD below the mean (Weak 2), those with less than one to two SD below the mean (Weak 3), those with less than two SD below the mean (Weak 4), and those stronger than the reference group. In the male sample, mean strength within the reference group was 43·9 kg, corresponding to about 3·9 kg more compared to the first weak group and about 17·1 kg more than the weakest group. In the female sample, mean strength within the reference group was 26·8 kg, corresponding to about 2·7 kg, more than the first weak group and about 12·0 kg more than the weakest group. Notably, the median age hardly varies between the HGS-groups, given the standardisation.

For peer review only - http://bmjopen.bmj.com/site/about/guidelines.xhtml

1	Groups	Strong	Reference	Weak 1	Weak 2	Weak 3	Weak 4
2	Thresholds	[0·5, 3·0)	[0·0, 0·5)	[-0·5, 0·0)	[-1·0, -0·5)	[-2·0, -1·0)	[-3·0, -2·0)
	**Men**						
3	Median age	68·2	67·9	68·8	68·5	67·6	66·2
4	Mean grip in kg (SD)	50·2 (5·8)	43·9 (4·5)	40·0 (4·6)	36·5 (4·7)	32·6 (4·7)	26·8 (4·6)
5	HR (95% CI)	0·93 (0·69, 1·25)	1·00	1·67 (1·23, 2·26)	2·02 (1·49, 2·75)	2·40 (1·77, 3·26)	2·34 (1·40, 3·93)
6	N	1142 (31·9%)	688 (19·2%)	647 (18·1%)	522 (14·6%)	481 (13·4%)	103 (2·9%)
	**Women**						
7	Median age	67·8	67·6	68·2	67·9	66·8	67·6
8	Mean grip in kg (SD)	30·8 (3·9)	26·8 (2·9)	24·1 (2·9)	21·9 (2·9)	19·1 (3·1)	14·8 (2·9)
9	HR (95% CI)	0·90 (0·66, 1·22)	1·00	1·32 (0·96, 1·82)	1·65 (1·20, 2·28)	1·85 (1·34, 2·55)	3·03 (1·83, 5·04)
10	N	1438 (31·4%)	901 (19·7%)	791 (17·3%)	695 (15·2%)	645 (14·1%)	103 (2·3%)

SD = standard deviation; CI = confidence intervals; N = number of observations; extended tables with years of education as a control are provided in Table A1 of the Supplementary Material

Table 2: Hazard ratios and sample characteristics by gender and st_HGS group

Statistical analysis

As a first step, we estimate the association between the six st_HGS groups (strong, reference, Weak 1-4) and all-cause mortality using separate Cox proportional hazard models for women and men. We control for age in the first model and add years of education for robustness analyses in a second model, to account for the strong correlation of education with the speed of ageing.[26] Since estimated HR are not informative in terms of life expectancy differences, in a second step, we provide RLE estimates at ages 60 and 70 for both genders. More specifically, we approximate RLE by estimating segmented life expectancy from age 60 to 90 as well as from age 70 to 90 based on a Gompertz model that includes age as a covariate. Hence, strictly speaking, RLE gives the average number a person lives between the ages 60 and 90, as opposed to the average number a person lives after age 60. This approach is necessary due to the small number of observations that live beyond age 90 in the cohorts observed and the HRS sample – there exclusion facilitates the prevention of biased estimates.

We applied bootstrapping, running 1,000 simulations for each gender, and report the median RLE for each of the six st_HGS groups. Finally, we provide straightforward data visualisations that allows for an easy assessment of the mortality risk group based on measured HGS in kg. All statistical analyses were conducted using R.

For peer review only

For peer review only - http://bmjopen.bmj.com/site/about/guidelines.xhtml

Patient and Public Involvement

There was no direct patient or public involvement in this research, as the analysis is based on secondary observational data.

RESULTS

Hazard ratios of mortality by HGS group and gender

Results from the Cox proportional hazard models suggest that survival already decreases just below group-specific average HGS, namely in groups Weak 1 and 2. HR of mortality by gender and across standardised HGS groups are presented in Table 2, Rows 5 and 9 (see also Table A1 in Supplementary Material). The comparison of the male reference group [0·0 SD, 0·5 SD) with three weaker groups suggests that men's mortality risk increases in a monotonous fashion with each weaker group. The risk already increases substantially when the standardised HGS falls into the Weak 1 group [-0·5 SD, 0·0 SD) with a HR 1·67 (95% CI 1·23–2·26). Subsequently, the HR further increases to 2·02 (1·49–2·75) in the Weak 2 group and to 2·40 (1·77–3·26) in the Weak 3 group. The weakest group [-3·0, -2·0) only comprised a small fraction of the sample (2·9%) and showed a HR of 2·34 with a large CI (1·40–3·93). The reference group and the strong group [0·5 SD, 3·0 SD) were similar in terms of mortality risks (men: HR 0·93, 95% CI 0·69–1·25; women: 0·90, 95% CI 0·66–1·22), indicating that there are no survival benefits associated with above-average HGS. The pattern of results for women is similar (Table 2, Row 9) but involved somewhat smaller HR that increased from 1·32 (95% CI 0·96–1·82) in Weak 1 to 1·65 (1·20–2·28) in Weak 2, 1·85 (1·34–2·55) in Weak 3, and 3·03 (1·83–5·04) in Weak 4. Sensitivity analyses controlling for educational attainment yielded virtually identical results (see Table A1 of the Supplementary Material). Survival curves and numbers at risk are shown in Figure A1 of the Supplementary Material.

Differences in remaining life expectancy by HGS thresholds and gender

Similar to the results from the Cox proportional hazard models, RLE shows how small negative deviations from the reference group have already substantial impact on life expectancy for both genders. Table 3 provides RLE and threshold values for each HGS group for two reference ages, namely ages 60 and 70, with average height (175 cm for men and 163 cm for women). For example, men at age 60 in the Weak 1 group [-0·5 SD, 0·0 SD) are estimated to have RLE of 18·4 years — about 3·0 years less compared to the reference group (21·4 years). Their counterparts in the Weak 3 group [-2·0 SD, -1·0 SD) have an estimated RLE of 16·2 years — 5·2 years less compared to the reference group. Group differences in RLE among women are somewhat smaller but still substantial. The Weak 3 group is, for example, estimated to have RLE of 20·5 at age 60, which is 3·3 years lower compared to the reference group (23·8 years).

For peer review only - http://bmjopen.bmj.com/site/about/guidelines.xhtml

Group-specific HGS thresholds in kg

Table 3 also provides group-specific threshold values in kg for two reference ages with average height, which further highlight how small negative deviations in HGS from the norm have a substantial impact on survival. For a 60-year-old man with average height of 175 cm, for example, those with HGS of less than 45·9 kg fall into the Weak 1 group, which already shows a substantially elevated mortality risk compared to the age-specific reference group that has HGS of 45·9 to 49·4 kg. Practitioners should thus already start looking into underlying risk factors when HGS falls short of the norm by a few kg, but certainly be concerned when HGS is 0·5 SD below the group-specific threshold.

Notably, for 70-year-old men with average height, these threshold values are substantially lower, e.g., the threshold for Weak 1 is 41·2 kg instead of 45·9 kg for the 60-year-olds, which underlines the claim that group-specific threshold values are required instead of absolute threshold values in kg when assessing risk of death.

Groups	Thresholds	Age 60		Age 70	
		Range in kg	RLE	Range in kg	RLE
**Men**					
Strong:	[0·5 SD, 3·0 SD)	[49·4, max)	21·8	[44·8, max)	15·4
Reference:	[0·0 SD, 0·5 SD)	[45·9, 49·4)	21·4	[41·2, 44·8)	15·1
Weak 1:	[-0·5 SD, 0·0 SD)	[42·3, 45·9)	18·4	[37·7, 41·2)	13·0
Weak 2:	[-1·0 SD, -0·5 SD)	[38·7, 42·3)	17·3	[34·1, 37·7)	12·1
Weak 3:	[-2·0 SD, -1·0 SD)	[31·6, 38·7)	16·2	[27·0, 34·1)	11·3
Weak 4:	[-3·0 SD, -2·0 SD)	[min, 31·6)	16·5	[min, 27·0)	11·5
**Women**					
Strong:	[0·5 SD, 3·0 SD)	[30·7, max)	24·3	[27·7, max)	16·5
Reference:	[0·0 SD, 0·5 SD)	[28·4, 30·7)	23·8	[25·4, 27·7)	16·2
Weak 1:	[-0·5 SD, 0·0 SD)	[26·0, 28·4)	22·4	[23·0, 25·4)	15·2
Weak 2:	[-1·0 SD, -0·5 SD)	[23·7, 26·0)	21·2	[20·6, 23·0)	14·4
Weak 3:	[-2·0 SD, -1·0 SD)	[18·9, 23·7)	20·5	[15·9, 20·6)	13·9
Weak 4:	[-3·0 SD, -2·0 SD)	[min, 18·9)	17·5	[min, 15·9)	11·7

Table 3: Threshold values in kg and remaining life expectancy (RLE) for average height (175 cm for men and 163 cm for women) and two reference ages (60 and 70) by gender and st_HGS group

Using the HGS thresholds to detect increasing mortality risk early on

Findings from the Cox proportional hazard model as well as RLE show that practitioners should start being concerned about underlying and potentially undiagnosed risks already when HGS is just below that of the reference group, as mortality risk increases already for the group [-0·5 SD, 0·0 SD). We provide visualisations that allow for a straight-forward assessment of survival based on our models in Figure 1. The graph directly links HGS in kg to the HGS thresholds most relevant for an early detection of increased mortality risk, namely the reference group, the Weak 1, and the Weak 2 group.

For peer review only - http://bmjopen.bmj.com/site/about/guidelines.xhtml

Age-specific thresholds are provided for a set of gender-height combinations, since differences in HGS (in kg) between genders and height-groups are not informative for mortality assessments. The graph shows, for example, that a man aged 70 with height 170 cm and a measured HGS of 40 kg would be above the threshold for the reference group (green line), thus not leading to concerns regarding increased mortality risks. A taller man of 175 cm, however, with same age and same measured HGS would fall into the Weak 1 group (above orange line), suggesting that a more thorough health screening and health-enhancing clinical interventions may be needed.

-- Figure 1 here --

Figure 1: Model-based HGS thresholds (lower bound) in kg by gender, age, and body height

DISCUSSION

This study aimed at providing HGS thresholds for the general population, which enable an early detection of increased mortality risks related to muscle weakness. The contributions made have wide applicability, as the cut-off points provided in this paper along with their visual illustration can be routinely implemented in medical practice, and their link to RLE much easier communicated to patients than previously reported HR. Hence, these thresholds can be used as a screening tool to identify patients that would benefit from further assessments, healthcare interventions, or lifestyle changes.

The majority of clinical cut-off values for defining a weak grip are given with HGS measured in kg, separately by gender, but irrespective of age and body height. To the best of our knowledge, this is the first study considering both the inherent, non-informative increase in muscle strength with body height as well as its decrease with age when providing HGS thresholds based on representative data. We advocate the use of z-standardised HGS in clinical practice, based on our results confirming that HGS varies substantially with gender, age, and height, calling for reference points that differentiate between these dimensions.

Our results show that deviations from the reference group matter only for HGS below the group-specific 'norm' (reference group), whereas superior HGS has no beneficial effect on survival. Moreover, we find that survival decreases monotonically with below-average HGS, preventing the use of log-rank or similar methods for identifying optimal cut-off points. Previous evidence regarding the shape of the association between HGS and mortality is mixed. A recent meta-analysis concludes that the relationship is linear,[5] while other studies suggest that high levels of HGS do not provide additional protection for mortality compared to medium levels,[27] thus supporting our findings.

For peer review only - http://bmjopen.bmj.com/site/about/guidelines.xhtml

From our findings it is clear that HGS, when standardised for gender, age, and body height, is a precise biomarker of (non)healthy ageing, indicative of an increased risk of death already when it deviates only half a SD from the norm, i.e., the respective reference group. For example, when comparing men aged 50 to 80 in the reference group [0·0 SD, 0·5 SD) with men that have the same age and body height but slightly weaker HGS [-0·5 SD, 0·0 SD), the latter were shown to be 67% more likely to die earlier (HR 1·67, 95% CI 1·23–2·26). Notably, this HR is similar to prior work that has used substantially lower HGS thresholds, i.e., included much weaker individuals in the risk group.[28] Using HRS data, Duchowny[29] defined muscle weakness as < 35 kg for Caucasian men and < 22 kg for women and showed that it is associated with a 50% greater risk of death over the follow-up period of nine years (HR=1.52, 95% CI 1.15-1.47). Again, these absolute thresholds are substantially higher than the ones suggested in our study. In the present paper, differences in HGS in kg between the group-specific reference and the group Weak 1, which has a HR of 1.67, are rather small, highlighting once again how exact HGS measurements are. As shown in Table 2, the difference in the average HGS for men between the reference group and the group Weak 1 is about 4 kg, and even smaller for women. This is also clear from Figure 1, where the threshold lines of each risk group are very tight.

Most of the limitations of this study are data driven. First, we only kept Caucasian individuals due to the small number of observations from other races, which is relevant since previous work has found substantial differences in reference HGS by race.[20] Second, it is possible that the frailest among the survey participants are not able to perform the HGS test. Previous work has shown that these individuals have, naturally, also higher mortality and their item non-response might potentially lead to an underestimation of mortality in our sample.[30]

Despite its data limitations, this study has confirmed that muscle strength is a powerful and precise predictor of survival, and HGS a quick and inexpensive way to assess reductions in RLE in clinical practices, as long as inherent variations by gender, age, and body height are considered in the cut-off points applied. In conclusion, survival decreases just below the gender-, age-, and height-specific HGS norms, which is why we suggest a cut-off point of 0.5 – or even smaller – to detect patients with an increased mortality risk early on.

For peer review only - http://bmjopen.bmj.com/site/about/guidelines.xhtml

ACKNOWLEDGEMENT

The research leading to these results has received funding from the European Research Council under the European Union's Seventh Framework Programme (FP7/2007–2013)/ERC grant agreement no ERC2012-AdG 323947-Re-Ageing.

ROLE OF THE FUNDING SOURCE

The funder of the study had no role in study design, data analysis, data interpretation, writing of the manuscript; or decision to submit the manuscript for publication. All authors are independent from the funder and had full access to all of the data in the study and thus can take responsibility for the integrity of the data and the accuracy of the data analysis.

DATA SHARING STATEMENT

The study used anonymised secondary data that are made available to all interested researchers upon request from the Health and Retirement Study.

ETHICS APPROVAL

This manuscript is based on secondary data from the Health and Retirement Study, which has been conducted according to the Declaration of Helsinki and was approved by the University of Michigan Institutional Review Board.

TRANSPARANCY STATEMENT

The corresponding author affirms that the manuscript is an honest, accurate, and transparent account of the study being reported, that no important aspects of the study have been omitted, and that any discrepancies from the study as originally planned have been explained.

REFERENCES

1 Clegg A, Young J, Iliffe S, Rikkert MO, Rockwood K. Frailty in elderly people. *Lancet Lond Engl* 2013; **381**: 752–62.
2 Fried LP, Tangen CM, Walston J, *et al.* Frailty in Older Adults Evidence for a Phenotype. *J Gerontol A Biol Sci Med Sci* 2001; **56**: M146–57.
3 Cruz-Jentoft AJ, Baeyens JP, Bauer JM, *et al.* Sarcopenia: European consensus on definition and diagnosis: Report of the European Working Group on Sarcopenia in Older People. *Age Ageing* 2010; **39**: 412–23.
4 Cruz-Jentoft AJ, Bahat G, Bauer J, *et al.* Sarcopenia: revised European consensus on definition and diagnosis. *Age Ageing* 2019; **48**: 16–31.
5 Wu Y, Wang W, Liu T, Zhang D. Association of Grip Strength With Risk of All-Cause Mortality, Cardiovascular Diseases, and Cancer in Community-Dwelling Populations: A Meta-analysis of Prospective Cohort Studies. *J Am Med Dir Assoc* 2017; **18**: 551.e17-551.e35.
6 Rijk JM, Roos PR, Deckx L, van den Akker M, Buntinx F. Prognostic value of handgrip strength in people aged 60 years and older: A systematic review and meta-analysis. *Geriatr Gerontol Int* 2015; published online May 28. DOI:10.1111/ggi.12508.
7 Mainous III AG, Tanner RJ, Anton SD, Jo A. Grip Strength as a Marker of Hypertension and Diabetes in Healthy Weight Adults. *Am J Prev Med* DOI:10.1016/j.amepre.2015.05.025.

For peer review only - http://bmjopen.bmj.com/site/about/guidelines.xhtml

8 Bhasin S, Travison TG, Manini TM, *et al.* Sarcopenia Definition: The Position Statements of the Sarcopenia Definition and Outcomes Consortium. *J Am Geriatr Soc* 2020; **68**: 1410–8.
9 Martin-Ruiz C, von Zglinicki T. Biomarkers of healthy ageing: expectations and validation. *Proc Nutr Soc* 2014; **73**: 422–9.
10 Leong DP, Teo KK, Rangarajan S, *et al.* Prognostic value of grip strength: findings from the Prospective Urban Rural Epidemiology (PURE) study. *The Lancet* 2015; **386**: 266–73.
11 Bohannon RW. Muscle strength: clinical and prognostic value of hand-grip dynamometry. *Curr Opin Clin Nutr Metab Care* 2015; **18**: 465–70.
12 Kuh D, Karunananthan S, Bergman H, Cooper R. A life-course approach to healthy ageing: maintaining physical capability. *Proc Nutr Soc* 2014; **73**: 237–48.
13 Lera L, Albala C, Leyton B, *et al.* Reference values of hand-grip dynamometry and the relationship between low strength and mortality in older Chileans. *Clin Interv Aging* 2018; **Volume 13**: 317–24.
14 Laukkanen JA, Voutilainen A, Kurl S, Araujo CGS, Jae SY, Kunutsor SK. Handgrip strength is inversely associated with fatal cardiovascular and all-cause mortality events. *Ann Med* 2020; **52**: 109–19.
15 Volaklis KA, Halle M, Meisinger C. Muscular strength as a strong predictor of mortality: A narrative review. *Eur J Intern Med* 2015; **26**: 303–10.
16 Zhuang C, Zhang F, Li W, *et al.* Associations of low handgrip strength with cancer mortality: a multicentre observational study. *J Cachexia Sarcopenia Muscle* 2020; **11**: 1476–86.
17 Xu X, Yang Z, Ma T, *et al.* The cut-off values of handgrip strength and lean mass index for sarcopenia among patients on peritoneal dialysis. *Nutr Metab* 2020; **17**: 84.
18 Alley DE, Shardell MD, Peters KW, *et al.* Grip strength cutpoints for the identification of clinically relevant weakness. *J Gerontol A Biol Sci Med Sci* 2014; **69**: 559–66.
19 McGrath R, Vincent BM, Peterson MD, *et al.* Weakness May Have a Causal Association With Early Mortality in Older Americans: A Matched Cohort Analysis. *J Am Med Dir Assoc* 2019; published online Nov 27. DOI:10.1016/j.jamda.2019.10.016.
20 Duchowny K, Peterson MD, Clarke P. Cut points for Clinical Muscle Weakness Among Older Americans. *Am J Prev Med* 2017; **53**: 63–9.
21 Steiber N. Strong or Weak Handgrip? Normative Reference Values for the German Population across the Life Course Stratified by Sex, Age, and Body Height. *PLOS ONE* 2016; **11**: e0163917.
22 Dodds RM, Syddall HE, Cooper R, *et al.* Grip Strength across the Life Course: Normative Data from Twelve British Studies. *PLoS ONE* 2014; **9**: e113637.
23 Kallman DA, Plato CC, Tobin JD. The role of muscle loss in the age-related decline of grip strength: cross-sectional and longitudinal perspectives. *J Gerontol* 1990; **45**: M82-88.
24 HRS. Health and Retirement Study, public use dataset. Produced and distributed by the University of Michigan with funding from the National Institute on Aging (grant number NIA U01AG009740). Ann Arbor, MI http://hrsonline.isr.umich.edu/. 2015.
25 Roberts HC, Denison HJ, Martin HJ, *et al.* A review of the measurement of grip strength in clinical and epidemiological studies: towards a standardised approach. *Age Ageing* 2011; **40**: 423–9.
26 Sanderson WC, Scherbov S. Measuring the Speed of Aging across Population Subgroups. *PLoS ONE* 2014; **9**: e96289.
27 Ortega FB, Silventoinen K, Tynelius P, Rasmussen F. Muscular strength in male adolescents and premature death: cohort study of one million participants. *The BMJ* 2012; **345**: e7279.
28 Cooper R, Kuh D, Hardy R, Mortality Review Group, on behalf of the FALCon and HALCyon study teams. Objectively measured physical capability levels and mortality: systematic review and meta-analysis. *BMJ* 2010; **341**: c4467–c4467.
29 Duchowny K. Do Nationally Representative Cutpoints for Clinical Muscle Weakness Predict Mortality? Results From 9 Years of Follow-up in the Health and Retirement Study. *J Gerontol Ser A* 2019; **74**: 1070–5.
30 Bachettini NP, Bielemann RM, Barbosa-Silva TG, Menezes AMB, Tomasi E, Gonzalez MC. Sarcopenia as a mortality predictor in community-dwelling older adults: a comparison of the diagnostic criteria of the European Working Group on Sarcopenia in Older People. *Eur J Clin Nutr* 2020; **74**: 573–80.

For peer review only - http://bmjopen.bmj.com/site/about/guidelines.xhtml

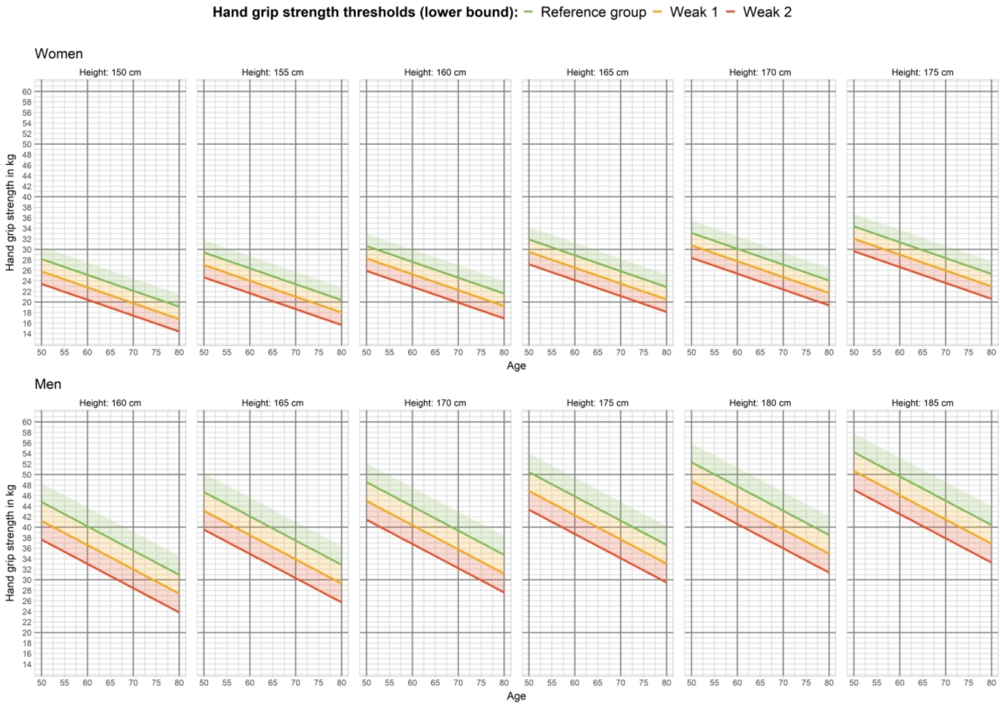

Figure 1: Model-based HGS thresholds (lower bound) in kg by gender, age, and body height

1398x1016mm (118 x 118 DPI)

SUPPLEMENTARY MATERIAL

	Model 1 (without education)	Model 2 (with education))
**Men**		
Reference: [0·0 SD, 0·5 SD)	1·00 (reference)	1·00 (reference)
Strong: [0·5 SD, 3·0 SD)	0·93 (0·69-1·25)	0·93 (0·69-1·26)
Weak 1: [-0·5 SD, 0·0 SD)	1·67 (1·23-2·26)	1·68 (1·24-2·27)
Weak 2: [-1·0 SD, -0·5 SD)	2·02 (1·49-2·75)	1·98 (1·46-2·69)
Weak 3: [-2·0 SD, -1·0 SD)	2·40 (1·77-3·26)	2·39 (1·76-3·25)
Weak 4: [-3·0 SD, -2·0 SD)	2·34 (1·40-3·93)	2·32 (1·39-3·89)
Age in years	1·09 (1·07-1·10)	1·09 (1·07-1·10)
Years of education		0·96 (0·93-0·98)
Max log likelihood	-2358·9	-2353·0
LR test statistic	244·6	256·4
Total time at risk	21864	21864
Events	529	529
**Women**		
Reference: [0·0 SD, 0·5 SD)	1·00 (reference)	1·00 (reference)
Strong: [0·5 SD, 3·0 SD)	0·90 (0·66-1·22)	0·89 (0·66-1·22)
Weak 1: [-0·5 SD, 0·0 SD)	1·32 (0·96-1·82)	1·30 (0·94-1·79)
Weak 2: [-1·0 SD, -0·5 SD)	1·65 (1·20-2·28)	1·62 (1·18-2·24)
Weak 3: [-2·0 SD, -1·0 SD)	1·85 (1·34-2·55)	1·83 (1·32-2·53)
Weak 4: [-3·0 SD, -2·0 SD)	3·03 (1·83-5·04)	2·98 (1·79-4·96)
Age in years	1·10 (1·08-1·12)	1·10 (1·08-1·11)
Years of education		0·93 (0·90-0·96)
Max log likelihood	-2192·8	-2181·7
LR test statistic	212·6	234·8
Total time at risk	28500	28500
Events	449	449

95% CI in parentheses, years of education < 20 years

Table A1: Hazard ratios for mortality by gender and st_HGS group, with and without years of education as control

For peer review only

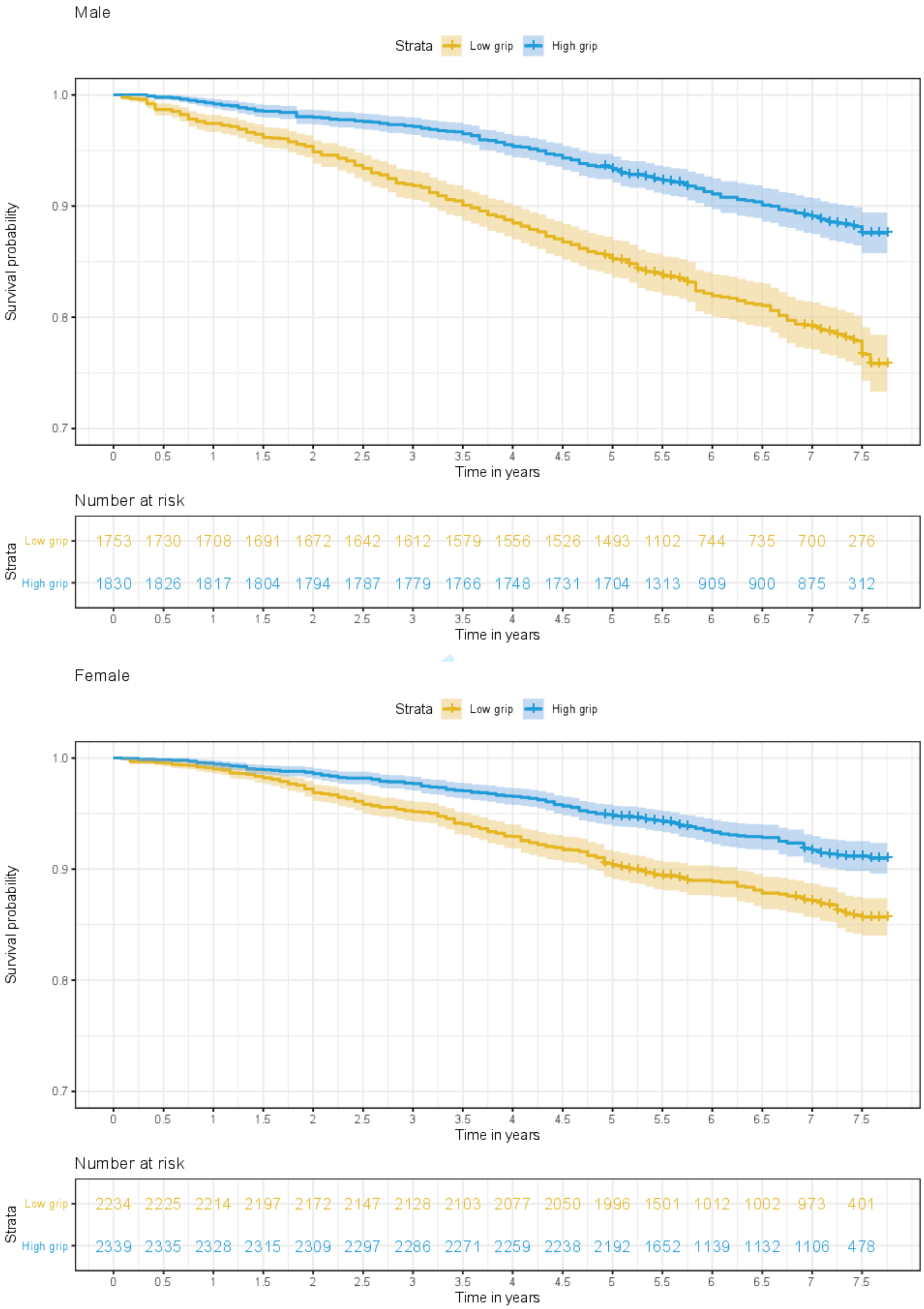

“High grip” refers to the strong and reference group and “low grip” to groups Weak1-4

Figure A1: Survival and numbers at risk, by gender and standardised handgrip strength

STROBE Statement—Checklist of items that should be included in reports of ***cross-sectional studies***

	Item No	Recommendation	Page No
**Title and abstract**	1	(*a*) Indicate the study's design with a commonly used term in the title or the abstract	2
		(*b*) Provide in the abstract an informative and balanced summary of what was done and what was found	2
**Introduction**			
Background/rationale	2	Explain the scientific background and rationale for the investigation being reported	2-5
Objectives	3	State specific objectives, including any prespecified hypotheses	2,5
**Methods**			
Study design	4	Present key elements of study design early in the paper	2,5
Setting	5	Describe the setting, locations, and relevant dates, including periods of recruitment, exposure, follow-up, and data collection	5-6
Participants	6	(*a*) Give the eligibility criteria, and the sources and methods of selection of participants	5-6
Variables	7	Clearly define all outcomes, exposures, predictors, potential confounders, and effect modifiers. Give diagnostic criteria, if applicable	5-8
Data sources/ measurement	8*	For each variable of interest, give sources of data and details of methods of assessment (measurement). Describe comparability of assessment methods if there is more than one group	*5-6*
Bias	9	Describe any efforts to address potential sources of bias	8
Study size	10	Explain how the study size was arrived at	5-6
Quantitative variables	11	Explain how quantitative variables were handled in the analyses. If applicable, describe which groupings were chosen and why	5-8
Statistical methods	12	(*a*) Describe all statistical methods, including those used to control for confounding	5-8
		(*b*) Describe any methods used to examine subgroups and interactions	5-8
		(*c*) Explain how missing data were addressed	5-8
		(*d*) If applicable, describe analytical methods taking account of sampling strategy	5-8
		(*e*) Describe any sensitivity analyses	5-8
**Results**			
Participants	13*	(a) Report numbers of individuals at each stage of study—eg numbers potentially eligible, examined for eligibility, confirmed eligible, included in the study, completing follow-up, and analysed	5-10
		(b) Give reasons for non-participation at each stage	5-10
		(c) Consider use of a flow diagram	NA
Descriptive data	14*	(a) Give characteristics of study participants (eg demographic, clinical, social) and information on exposures and potential confounders	5-10
		(b) Indicate number of participants with missing data for each variable of interest	NA
Outcome data	15*	Report numbers of outcome events or summary measures	5-10
Main results	16	(*a*) Give unadjusted estimates and, if applicable, confounder-adjusted estimates and their precision (eg, 95% confidence interval). Make clear which confounders were adjusted for and why they were included	2-13

For peer review only - http://bmjopen.bmj.com/site/about/guidelines.xhtml

		(*b*) Report category boundaries when continuous variables were categorized	NA
		(*c*) If relevant, consider translating estimates of relative risk into absolute risk for a meaningful time period	NA
Other analyses	17	Report other analyses done—eg analyses of subgroups and interactions, and sensitivity analyses	5-8
**Discussion**			
Key results	18	Summarise key results with reference to study objectives	8-13
Limitations	19	Discuss limitations of the study, taking into account sources of potential bias or imprecision. Discuss both direction and magnitude of any potential bias	12-13
Interpretation	20	Give a cautious overall interpretation of results considering objectives, limitations, multiplicity of analyses, results from similar studies, and other relevant evidence	12-13
Generalisability	21	Discuss the generalisability (external validity) of the study results	12-13
**Other information**			
Funding	22	Give the source of funding and the role of the funders for the present study and, if applicable, for the original study on which the present article is based	13

*Give information separately for exposed and unexposed groups.

Note: An Explanation and Elaboration article discusses each checklist item and gives methodological background and published examples of transparent reporting. The STROBE checklist is best used in conjunction with this article (freely available on the Web sites of PLoS Medicine at http://www.plosmedicine.org/, Annals of Internal Medicine at http://www.annals.org/, and Epidemiology at http://www.epidem.com/). Information on the STROBE Initiative is available at www.strobe-statement.org.

For peer review only

BMJ Open

Thresholds for clinical practice that directly link handgrip strength to remaining years of life: Estimates based on longitudinal observational data

Journal:	*BMJ Open*
Manuscript ID	bmjopen-2021-058489.R1
Article Type:	Original research
Date Submitted by the Author:	22-Apr-2022
Complete List of Authors:	Scherbov, Sergei; International Institute for Applied Systems Analysis<br>Spitzer, Sonja; University of Vienna, Department of Demography<br>Steiber, Nadia; University of Vienna, Department of Sociology; Institute for Advanced Studies, Department of Sociology
**Primary Subject Heading**:	Geriatric medicine
Secondary Subject Heading:	General practice / Family practice, Diagnostics
Keywords:	GERIATRIC MEDICINE, PUBLIC HEALTH, STATISTICS & RESEARCH METHODS

SCHOLARONE™
Manuscripts

I, the Submitting Author has the right to grant and does grant on behalf of all authors of the Work (as defined in the below author licence), an exclusive licence and/or a non-exclusive licence for contributions from authors who are: i) UK Crown employees; ii) where BMJ has agreed a CC-BY licence shall apply, and/or iii) in accordance with the terms applicable for US Federal Government officers or employees acting as part of their official duties; on a worldwide, perpetual, irrevocable, royalty-free basis to BMJ Publishing Group Ltd ("BMJ") its licensees and where the relevant Journal is co-owned by BMJ to the co-owners of the Journal, to publish the Work in this journal and any other BMJ products and to exploit all rights, as set out in our licence.

The Submitting Author accepts and understands that any supply made under these terms is made by BMJ to the Submitting Author unless you are acting as an employee on behalf of your employer or a postgraduate student of an affiliated institution which is paying any applicable article publishing charge ("APC") for Open Access articles. Where the Submitting Author wishes to make the Work available on an Open Access basis (and intends to pay the relevant APC), the terms of reuse of such Open Access shall be governed by a Creative Commons licence – details of these licences and which Creative Commons licence will apply to this Work are set out in our licence referred to above.

Other than as permitted in any relevant BMJ Author's Self Archiving Policies, I confirm this Work has not been accepted for publication elsewhere, is not being considered for publication elsewhere and does not duplicate material already published. I confirm all authors consent to publication of this Work and authorise the granting of this licence.

For peer review only

TITLE PAGE

Thresholds for clinical practice that directly link handgrip strength to remaining years of life: Estimates based on longitudinal observational data

Dr. Sergei Scherbov

Principal Research Scholar, Population and Just Societies Program, International Institute for Applied Systems Analysis (IIASA), Wittgenstein Centre for Demography and Global Human Capital (IIASA, OeAW, University of Vienna), Schlossplatz 1, 2361 Laxenburg, Austria

E-mail: scherbov@iiasa.ac.at

Dr. Sonja Spitzer

Postdoctoral Researcher, Department of Demography, University of Vienna, Wittgenstein Centre for Demography and Global Human Capital (IIASA, OeAW, University of Vienna), Vordere Zollamtsstraße 3, 1030 Vienna, Austria

E-mail: sonja.spitzer@univie.ac.at

Dr. Nadia Steiber (corresponding author)

Professor, Department of Sociology, University of Vienna, Rooseveltplatz 2, 1090 Vienna, Austria and Institute for Advanced Studies, Vienna, Austria

E-mail: nadia.steiber@univie.ac.at

Phone: +43 1 4277 49290

AUTHOR CONTRIBUTIONS

SSC and NS designed the study, SSC was responsible for data curation and the statistical analyses, NS wrote the original draft, and SSP was responsible for reviewing and editing the manuscript and for data visualisation. All authors have verified the underlying data and are responsible for the overall content. The corresponding author attests that all listed authors meet authorship criteria and that no others meeting the criteria have been omitted.

PATIENT CONSENT

Not applicable

DECLARATION OF INTEREST

The authors declare no competing interests

WORD COUNT: 4,047 (including tables)

For peer review only - http://bmjopen.bmj.com/site/about/guidelines.xhtml

ABSTRACT

Objective: Muscle strength is a powerful predictor of mortality that can quickly and inexpensively be assessed by measuring handgrip strength (HGS). What is missing for clinical practice, however, are empirically meaningful cut-off points that apply to the general population, and that consider the correlation of HGS with gender and body height as well as the decline in HGS during processes of *normal* ageing. This study provides standardised thresholds that directly link HGS to remaining life expectancy (RLE), thus enabling practitioners to detect patients with an increased mortality risk early on.

Design: Relying on representative observational data from the Health and Retirement Study, the HGS of survey participants was z-standardised by gender, age, and body height. We defined six HGS groups based on cut-off points in standard deviation (SD); we use these as predictors in survival analyses with a 9-year follow-up and provide RLE by gender based on a Gompertz model for each HGS group.

Participants: 8,156 U.S. American women and men aged 50 to 80

Main outcome measures: z-standardised HGS and all-cause mortality

Results: Even slight negative deviations in HGS from the reference group with [0.0 SD, 0.5 SD) have substantial effects on survival. RLE among 60-year-olds with standardised HGS of [-0.5 SD, 0.0 SD) is 3.0/1.4 years lower for men/women than for the reference group, increasing to a difference of 4.1/2.6 years in the group with HGS of [-1.0 SD, -0.5 SD). By contrast, we find no benefit of strong HGS related to survival.

Conclusions: HGS varies substantially with gender, age, and body height. This confirms the importance of considering these heterogeneities when defining reference groups and risk thresholds. Moreover, survival appears to decrease at much higher levels of muscle strength than is assumed in previous literature, suggesting that medical practitioners should start to become concerned when HGS is slightly below that of the reference group.

For peer review only

For peer review only - http://bmjopen.bmj.com/site/about/guidelines.xhtml

Strengths and limitations of this study:

- We develop a clinical definition of low handgrip strength related to increased mortality risk and provide cut-off points, along with straightforward visualisations, that can be used in medical practice.
- In addition to hazard ratios, we present, for the first time, estimates of remaining life expectancy related to the cut-off points, enabling a much more intuitive interpretation of mortality risk that can easily be communicated to patients.
- Previous work suggests substantial differences in reference handgrip strength by race. This study provides cut-offs based on data from Caucasian individuals only, due to the small number of observations of HGS from other races.

INTRODUCTION

Muscle strength, as frequently measured by handgrip strength (HGS), is a well-established indicator of functional status that has been widely used as a key component of frailty phenotypes[1,2] and in diagnosing sarcopenia.[3,4] Beyond the use of HGS measurement in gerontological assessments, there is a large and growing body of research concerned with its prognostic value for future health conditions,[5] indicating that HGS is a powerful predictor of adverse health outcomes such as disability, cognitive decline, and eventually mortality.[6–8]

Given its strong association with the functional status of older individuals and its high prognostic value for future morbidity and mortality, HGS has been promoted as a biomarker of healthy ageing. It has been shown to be a better predictor of survival and the maintenance of good health than chronological age[9] and can be used as a screening tool for the vulnerability of older individuals that is much simpler and more cost-effective than comprehensive geriatric assessments.[10,11]

While there is consensus that HGS could be used routinely to screen for people whose future health is at risk and who may require intervention, research on empirically relevant threshold values for such screenings is scarce.[12] Recent studies on the predictive value of HGS for mortality have used either a continuous measure of HGS in kilograms (kg), or broad and arbitrary classifications of HGS into quantiles.[6,12–15] Moreover, studies are often based on small, non-representative samples that are targeted at patients suffering from certain diseases only.[16,17] Some studies use mediating health outcomes to define thresholds. The Foundation of the National Institutes of Health,[18] for example, constructed cut-off points (in kg) based on the statistical associations between HGS and low gait speed, which were used as mortality predictors in a subsequent study.[19]

For peer review only - http://bmjopen.bmj.com/site/about/guidelines.xhtml

The most popular threshold for weak HGS—also frequently used today to predict survival—is not actually conceptualised with mortality in mind but, instead, for the diagnosis of sarcopenia. In 2010 the European Working Group on Sarcopenia in Older Persons (EWGSOP) recommended a cut-off at two standard deviations (SD) below the mean value of HGS taken from a normative, healthy reference population to define muscle weakness.[3] In 2019 the group updated their thresholds, now suggesting a cut-off at 2.5 SD to define low HGS, translating to <27 kg for men and <16 kg for women.[4] This simple suggestion has become a common criterion, used worldwide, for the diagnosis of sarcopenia in geriatric assessment and gerontological research. Moreover, it is used in numerous studies as a threshold to predict mortality differentials.

However, when thresholds are defined as 2 or 2.5 SD below the mean value of a healthy reference population (i.e., the t-scores), the decline in HGS during processes of *normal* ageing from age 30 to 40 onwards[20]—which is associated with changing levels of physical activity, anabolic responsiveness to protein intake, and hormonal status[21]—cannot be accounted for. This decline is not indicative of pathological ageing, in other words, it is not associated with mortality risks higher than those seen in other individuals of the same chronological age. Using a threshold based on t-scores would imply that the majority of older individuals are at a higher risk of dying, and would simply reflect a lower life expectancy on the part of the old rather than provide an effective tool to detect the most vulnerable within each age group. Moreover, HGS increases substantially with body height,[20] which is an indicator neither of the absence of sarcopenia nor of higher survival. Adjusting HGS for body height thus has the potential to improve mortality risk assessments.[14] In a similar vein, women have a lower average HGS than men,[22] and HGS varies by race.[23]

In their most recent report, EWGSOP thus highlights the need for studies establishing gender- and region-specific threshold values to improve outcome prediction.[4] Going one step further and accounting for processes of *normal* ageing and the association of HGS with anthropometric traits, we propose standardised threshold values that consider the inherent, non-informative variations in muscle strength with gender, age, and body height, i.e., z-scores, using separate reference categories for each gender–age–height group. The use of these threshold values enables practitioners to clearly detect patients who deviate from the norm (i.e., their reference group) and who would thus have a higher, or potentially lower, risk of death.

The aim of the present study is thus to define standardised threshold values for HGS that are associated with a substantially increasing risk of mortality, using z-scores. We provide estimates based on representative data for U.S. Americans aged 50 and above, irrespective of their health status, and directly link muscle strength to mortality, without any deviations to mediating diseases. In addition to defining standardised thresholds, we go beyond previous work by providing estimates of remaining life

For peer review only - http://bmjopen.bmj.com/site/about/guidelines.xhtml

expectancy (RLE) associated with each of the cut-off points. In contrast to hazard ratios (HR), RLE provides information about actual life expectancy differences between HGS groups and can be communicated to patients more intuitively. Moreover, we produce clear illustrations of the relationship between tested HGS and the risk groups, something which can routinely be used in medical practice as a low-entry starting point for further geriatric assessments and health-enhancing patient interventions.

METHODS

Data, sample, and study design

Our analysis is based on the Health and Retirement Study (HRS), a longitudinal panel survey providing representative data for the U.S. American population aged 50 and older.[24] In 2006 half the survey participants were randomly selected to perform an HGS test, with the other half completing the test in 2008. HRS can be linked to mortality register data from the National Death Index, and this allowed us to follow survey participants until December 2014. Participants can be linked to the National Death Index even if they leave the study early on, making attrition of minor concern for this analysis.

We restrict our sample to individuals aged 50 to 80, keeping all observations of HGS of at least 6 kg. To remove outliers in terms of anthropometric traits, we only keep women with a body height of 130 to 190 cm and men who are at least 150 cm in height. We focus on Caucasian individuals, as previous work shows a need for separate HGS thresholds by race,[23] and the number of observations for the other ethnic groups was too small for heterogeneity analyses. Finally, we drop cases with residuals larger than 2.5 SD from gender-specific linear regressions of age and body height on HGS (see next subsection for details). This leaves us with a sample of 8,156 individuals to predict death with a follow-up period of up to 9 years, during which 978 participants died. More detailed sample characteristics are provided in Table 1, and a chart visualising the sample construction can be found in the Supplementary Material (Figure A1).

For peer review only

Table 1: Participant characteristics stratified by gender

	Men	Women
Grip strength, kg, mean (SD)	42.1 (8.4)	25.5 (5.5)
Death during follow-up	529 (14.8%)	449 (9.8%)
Age, years, median (IQR)	68.2 (61.8–73.3)	67.7 (61.6–73.1)
Height, cm, median (IQR)	174.6 (170.2–179.1)	160.0 (156.2–164.5)
N	3,583	4,573

SD = standard deviation; IQR = interquartile range 75%–25%

For peer review only - http://bmjopen.bmj.com/site/about/guidelines.xhtml

Handgrip strength measurement and z-standardisation by gender, age, and body height

HGS was measured using a Smedley spring-type hand dynamometer. Two measurements were taken from each hand. Following published recommendations,[25] we used the maximum value achieved with either hand for our analyses.

As mentioned above, there is a strong variation in HGS by gender, age, and body height that is not directly linked to differences in mortality risks. We thus use a straightforward regression technique to adjust HGS values for these characteristics. First, we regress HGS on age and body height, using separate linear models for women and men. Squared age and height terms were not significant. Regression analyses confirm the strong differences in HGS by gender, age, and body height. More specifically, the expected HGS at baseline in 2006/2008 at ages 50 to 80 can be expressed as:

Men: $HGS_m = 7.23 + 0.38 \times height(cm) - 0.46 \times age(yr)$ (Adjusted R2: 0.28)

Women: $HGS_f = 6.00 + 0.25 \times height(cm) - 0.30 \times age(yr)$ (Adjusted R2: 0.27)

Second, we calculate the standardised residuals from the above equations, applying a z-standardisation with a mean of 0 and SD of 1 to obtain a measurement of HGS for each observation that is standardised for gender, age, and body height, denoted in the following as st_HGS_i. This allows for the construction of reference categories for each gender–age–height combination. Deviations in st_HGS_i from these reference categories enable mortality risk differentials to be detected.

In a third step we define thresholds that allow for early detection of patients with an increased mortality risk. Attempts at defining optimal cut-off points using a log-rank or similar method produced non-robust results due to the monotonic increase in the mortality risk with st_HGS_i. We thus construct six groups based on SD thresholds, as shown in Table 2, Rows 1 and 2. Given the high prevalence of chronic diseases in the study population, which is aged 50 and above, we defined the "healthy" reference group as those with st_HGS_i at or slightly above the mean [0.0 SD, 0.5 SD). The comparison groups comprise those with up to 0.5 SD below the mean (Weak 1), those with less than a half to a full SD below the mean (Weak 2), those with less than one to two SD below the mean (Weak 3), those with less than two SD below the mean (Weak 4), and those stronger than the reference group (Strong). In the male sample, mean strength within the reference group was 43.9 kg, corresponding to about 3.9 kg more than the first weak group and about 17.1 kg more than the weakest group. In the female sample, mean strength within the reference group was 26.8 kg, corresponding to about 2.7 kg, more than the first weak group and about 12.0 kg more than the weakest group. Notably, the median age hardly varies between the HGS groups, given the standardisation.

For peer review only - http://bmjopen.bmj.com/site/about/guidelines.xhtml

Table 2: Hazard ratios and sample characteristics by gender and st_HGS group

1	Groups	Strong	Reference	Weak 1	Weak 2	Weak 3	Weak 4
2	Thresholds	[0.5, 3.0)	[0.0, 0.5)	[-0.5, 0.0)	[-1.0, -0.5)	[-2.0, -1.0)	[-3.0, -2.0)
	**Men**						
3	Median age	68.2	67.9	68.8	68.5	67.6	66.2
4	Mean grip in kg (SD)	50.2 (5.8)	43.9 (4.5)	40.0 (4.6)	36.5 (4.7)	32.6 (4.7)	26.8 (4.6)
5	HR (95% CI)	0.93 (0.69, 1.25)	1.00	1.67 (1.23, 2.26)	2.02 (1.49, 2.75)	2.40 (1.77, 3.26)	2.34 (1.40, 3.93)
6	N	1142 (31.9%)	688 (19.2%)	647 (18.1%)	522 (14.6%)	481 (13.4%)	103 (2.9%)
	**Women**						
7	Median age	67.8	67.6	68.2	67.9	66.8	67.6
8	Mean grip in kg (SD)	30.8 (3.9)	26.8 (2.9)	24.1 (2.9)	21.9 (2.9)	19.1 (3.1)	14.8 (2.9)
9	HR (95% CI)	0.90 (0.66, 1.22)	1.00	1.32 (0.96, 1.82)	1.65 (1.20, 2.28)	1.85 (1.34, 2.55)	3.03 (0.83, 5.04)
10	N	1438 (31.4%)	901 (19.7%)	791 (17.3%)	695 (15.2%)	645 (14.1%)	103 (2.3%)

SD = standard deviation; CI = confidence intervals; N = number of observations; extended tables with years of education as a control are provided in Table A1 of the Supplementary Material

Statistical analysis

As a first step, we estimate the association between the six st_HGS groups (Strong, Reference, Weak 1–4) and all-cause mortality, using separate Cox proportional hazard models for women and men. We control for age in the first model and add years of education for robustness analyses in a second model to account for the strong correlation of education with the speed of ageing.[26] As the estimated HR are not informative in terms of differences in life expectancy, in a second step, we provide RLE estimates at age 60 and 70 for both genders. More specifically, we approximate RLE by estimating segmented life expectancy from age 60 to 90 and from age 70 to 90 based on a Gompertz model that includes age as a covariate. Hence, strictly speaking, RLE gives the average number of years a person lives between the ages 60 and 90, as opposed to after 60. This approach is necessary because of the small number of people living beyond the age of 90 in the cohorts observed and in the HRS sample—excluding them helps prevent biased estimates.

We applied bootstrapping, running 1,000 simulations for each gender, and report the median RLE for each of the 6 st_HGS groups. Finally, we provide straightforward data visualisations that allow for an easy assessment of the mortality risk associated with HGS measured in kg in conjunction with patients' age and body height. All statistical analyses were conducted using R.

For peer review only

Patient and Public Involvement

There was no direct patient or public involvement in this research, as the analysis is based on secondary observational data.

RESULTS

Hazard ratios of mortality by HGS group and gender

Results from the Cox proportional hazard models suggest that survival starts decreasing just below the group-specific average HGS, namely in groups Weak 1 and 2. The HR of mortality by gender and across standardised HGS groups are presented in Table 2, Rows 5 and 9 (see also Table A1 in Supplementary Material). The comparison of the male reference group [0.0 SD, 0.5 SD) with three weaker groups suggests that men's mortality risk increases in a monotonous fashion with each weaker group. The risk increases substantially when the standardised HGS falls into the Weak 1 group [-0.5 SD, 0.0 SD) with an HR of 1.67 (95% CI 1.23–2.26). Subsequently, the HR further increases to 2.02 (1.49–2.75) in the Weak 2 group and to 2.40 (1.77–3.26) in the Weak 3 group. The weakest group [-3.0, -2.0) comprised only a small fraction of the sample (2.9%) and showed an HR of 2.34 with a large CI (1.40–3.93). The reference group and the Strong group [0.5 SD, 3.0 SD) were similar in terms of mortality risks (men: HR 0.93, 95% CI 0.69–1.25; women: 0.90, 95% CI 0.66–1.22), indicating that there are no survival benefits associated with above-average HGS. The pattern of results for women is similar (Table 2, Row 9) but involved a somewhat smaller HR that increased from 1.32 (95% CI 0.96–1.82) in Weak 1 to 1.65 (1.20–2.28) in Weak 2, 1.85 (1.34–2.55) in Weak 3, and 3.03 (1.83–5.04) in Weak 4. Sensitivity analyses controlling for educational attainment yielded virtually identical results (see Table A1 of the Supplementary Material). Survival curves and numbers at risk are shown in Figure A2 of the Supplementary Material.

Differences in remaining life expectancy by HGS thresholds and gender

Similar to the results from the Cox proportional hazard models, RLE shows how small negative deviations from the reference group have substantial impact on life expectancy for both genders. Table 3 provides RLE and threshold values for each HGS group for two reference ages, namely 60 and 70, with average height being 175 cm for men and 163 cm for women. For example, men aged 60 in the Weak 1 group [-0.5 SD, 0.0 SD) are estimated to have an RLE of 18.4 years — about 3.0 years less than the reference group (21.4 years). Their counterparts in the Weak 3 group [-2.0 SD, -1.0 SD) have an estimated RLE of 16.2 years—5.2 years less than the reference group. Group differences in RLE among women are somewhat smaller but still substantial. The Weak 3 group is, for example, estimated to have an RLE of 20.5 at age 60, which is 3.3 years lower than that of the reference group (23.8 years).

For peer review only - http://bmjopen.bmj.com/site/about/guidelines.xhtml

Group-specific HGS thresholds in kg

Table 3 also provides group-specific threshold values in kg for two reference ages with average height, which further highlight how even small negative deviations in HGS from the norm have a substantial impact on survival. For a 60-year-old man with an average height of 175 cm, for example, those with HGS of less than 45.9 kg fall into the Weak 1 group, which already shows a substantially elevated mortality risk compared to the age-specific reference group, which has an HGS of 45.9 to 49.4 kg. Thus, practitioners should be looking into underlying risk factors when the HGS falls short of the norm by a few kg, but should certainly be concerned when the HGS is 0.5 SD below the group-specific threshold.

Notably, for 70-year-old men of average height, these threshold values are substantially lower, for example, the threshold for Weak 1 is 41.2 kg instead of 45.9 kg for the 60-year-olds, which underlines the claim that group-specific threshold values are required instead of absolute threshold values in kg when assessing health vulnerabilities and the risk of death.

Table 3: Threshold values in kg and remaining life expectancy (RLE) for average height (175 cm for men and 163 cm for women) and two reference ages (60 and 70) by gender and st_HGS group

Groups	Thresholds	Age 60		Age 70	
		Range in kg	RLE	Range in kg	RLE
**Men**					
Strong:	[0.5 SD, 3.0 SD)	[49.4, max)	21.8	[44.8, max)	15.4
Reference:	[0.0 SD, 0.5 SD)	[45.9, 49.4)	21.4	[41.2, 44.8)	15.1
Weak 1:	[-0.5 SD, 0.0 SD)	[42.3, 45.9)	18.4	[37.7, 41.2)	13.0
Weak 2:	[-1.0 SD, -0.5 SD)	[38.7, 42.3)	17.3	[34.1, 37.7)	12.1
Weak 3:	[-2.0 SD, -1.0 SD)	[31.6, 38.7)	16.2	[27.0, 34.1)	11.3
Weak 4:	[-3.0 SD, -2.0 SD)	[min, 31.6)	16.5	[min, 27.0)	11.5
**Women**					
Strong:	[0.5 SD, 3.0 SD)	[30.7, max)	24.3	[27.7, max)	16.5
Reference:	[0.0 SD, 0.5 SD)	[28.4, 30.7)	23.8	[25.4, 27.7)	16.2
Weak 1:	[-0.5 SD, 0.0 SD)	[26.0, 28.4)	22.4	[23.0, 25.4)	15.2
Weak 2:	[-1.0 SD, -0.5 SD)	[23.7, 26.0)	21.2	[20.6, 23.0)	14.4
Weak 3:	[-2.0 SD, -1.0 SD)	[18.9, 23.7)	20.5	[15.9, 20.6)	13.9
Weak 4:	[-3.0 SD, -2.0 SD)	[min, 18.9)	17.5	[min, 15.9)	11.7

Using the HGS thresholds to detect increasing mortality risk early on

Findings from the Cox proportional hazard model and also the RLE show that practitioners should start being concerned about underlying and potentially undiagnosed risks when the HGS is just below that of the reference group, as mortality risk increases already for the group [-0.5 SD, 0.0 SD). In Figure 1 we provide visualisations that allow for a straightforward assessment of survival based on our models. The graph directly links HGS in kg to the HGS thresholds most relevant for an early detection of increased mortality risk, namely the reference group, the Weak 1 group, and the Weak 2 group.

For peer review only - http://bmjopen.bmj.com/site/about/guidelines.xhtml

Age-specific thresholds are provided for a set of gender–height combinations, given that differences in HGS (in kg) between genders and height groups are not informative for mortality assessments. The graph shows, for example, that a man aged 70 with a height of 170 cm and a measured HGS of 40 kg would be above the threshold for the reference group (green line), and this would not lead to concerns regarding increased mortality risks. A taller man of 175 cm, however, with same age and same measured HGS would fall into the Weak 1 group (above orange line), suggesting that a more thorough health screening and health-enhancing clinical interventions may be needed.

-- Figure 1 here --

Figure 1: Model-based HGS thresholds (lower bound) in kg by gender, age, and body height. The reference group refers to HGS at or slightly above the mean [0.0 SD, 0.5 SD), Weak 1 to HGS up to 0.5 SD below the mean [-0.5 SD, 0.0 SD), and Weak 2 to less than a half to a full SD below the mean [-1.0 SD, -0.5 SD).

DISCUSSION

This study aimed to provide HGS thresholds for the general population to enable early detection of increased mortality risks related to muscle weakness. The contributions made have wide applicability, given that the cut-off points provided in this paper, along with a visual illustration of them, can be routinely implemented in medical practice and that their link to RLE can be communicated to patients much more easily than previously reported HR. These thresholds can thus be used as a screening tool to identify patients who would benefit from further assessments, healthcare interventions, or lifestyle changes.

The majority of clinical cut-off values for defining a weak grip are given by the HGS measured in kg, separately by gender, but irrespective of age and body height. To the best of our knowledge, this is the first study to provide HGS thresholds based on representative data that take into account both the inherently non-informative increase in muscle strength in conjunction with body height, and the decrease in HGS with age. We advocate the use of z-standardised HGS in clinical practice, based on our results confirming that HGS varies substantially with gender, age, and height, which calls for reference points that differentiate between these dimensions.

Our results show that deviations from the reference group matter only for HGS below the group-specific "norm" (reference group), whereas superior HGS has no beneficial effect on survival. Moreover, we find that survival decreases monotonically with below-average HGS, preventing the use of log-rank or similar methods for identifying optimal cut-off points. Previous evidence regarding the shape of the association between HGS and mortality is mixed. A recent meta-analysis concludes that the relationship is linear,[5] while other studies suggest that high levels of HGS do not provide additional protection for mortality compared to medium levels,[27] thus supporting our findings.

For peer review only - http://bmjopen.bmj.com/site/about/guidelines.xhtml
For peer review only

From our findings it is clear that HGS, when standardised for gender, age, and body height, is a precise biomarker of (non-)healthy ageing and indicative of an increased risk of death, even if it deviates by only half an SD from the norm (i.e., the respective reference group). For example, when comparing men aged 50 to 80 in the reference group [0.0 SD, 0.5 SD) with men of the same age and body height but with slightly weaker HGS [-0.5 SD, 0.0 SD), the latter were shown to be 67% more likely to die earlier (HR 1.67, 95% CI 1.23–2.26). Notably, this HR is similar to prior work that has used substantially lower HGS thresholds, (i.e., that included much weaker individuals in the risk group).[28] Using HRS data, Duchowny[29] defined muscle weakness as <35 kg for Caucasian men and <22 kg for women and showed it to be associated with a 50% greater risk of death over the follow-up period of 9 years (HR=1.52, 95% CI 1.15-1.47). Again, these absolute thresholds are substantially higher than the ones suggested in our study. In the present paper, differences in HGS in kg between the group-specific reference and the group Weak 1, which has a HR of 1.67, are quite small, highlighting once again how exact HGS measurements are as a screening tool. As shown in Table 2, the difference in the average HGS for men between the reference group and the group Weak 1 is about 4 kg, and even smaller for women. This is also clear from Figure 1, where the threshold lines of each risk group are very tight.

Most of the limitations of this study are data-driven. First, observations were made only for Caucasian individuals due to the small number of observations from other races; this is relevant given that previous studies have found substantial differences in reference HGS by race.[23] Second, the frailest of the survey participants may not be able to perform the HGS test. Previous studies have shown that these individuals also have naturally higher mortality and their non-response in this case could lead to an underestimation of mortality in our sample.[30] Our analysis is thus aimed at patients above a certain strength- and health-related threshold, who are able to perform the HGS test when consulting a practitioner and are, analogously, able to perform the HGS test when participating in a survey.

Despite its data limitations, this study has confirmed that muscle strength is a powerful and precise predictor of survival and that HGS is a quick and inexpensive way of assessing reductions in RLE in clinical practice, as long as inherent variations by gender, age, and body height are considered in the cut-off points applied. Our evidence points towards a threshold value for defining a critically weak handgrip that is much higher than that assumed in gerontological research to date. Survival decreases just below the gender-, age-, and height-specific HGS norms, which is why we suggest a cut-off point of 0.5—or even smaller—to detect patients with an increased mortality risk early on.

For peer review only - http://bmjopen.bmj.com/site/about/guidelines.xhtml

ACKNOWLEDGMENTS

Open access funding provided by University of Vienna.

ROLE OF THE FUNDING SOURCE

The funder of the study had no role in study design, data analysis, data interpretation, writing of the manuscript; or decision to submit the manuscript for publication. All authors are independent from the funder and had full access to all of the data in the study and thus can take responsibility for the integrity of the data and the accuracy of the data analysis.

DATA SHARING STATEMENT

The study used anonymised secondary data that are made available to all interested researchers upon request from the Health and Retirement Study.

ETHICS APPROVAL

This manuscript is based on secondary data from the Health and Retirement Study, which has been conducted according to the Declaration of Helsinki and was approved by the University of Michigan Institutional Review Board.

TRANSPARANCY STATEMENT

The corresponding author affirms that the manuscript is an honest, accurate, and transparent account of the study being reported, that no important aspects of the study have been omitted, and that any discrepancies from the study as originally planned have been explained.

REFERENCES

1 Clegg A, Young J, Iliffe S, Rikkert MO, Rockwood K. Frailty in elderly people. *Lancet Lond Engl* 2013; **381**: 752–62.

2 Fried LP, Tangen CM, Walston J, *et al.* Frailty in Older Adults Evidence for a Phenotype. *J Gerontol A Biol Sci Med Sci* 2001; **56**: M146–57.

3 Cruz-Jentoft AJ, Baeyens JP, Bauer JM, *et al.* Sarcopenia: European consensus on definition and diagnosis: Report of the European Working Group on Sarcopenia in Older People. *Age Ageing* 2010; **39**: 412–23.

4 Cruz-Jentoft AJ, Bahat G, Bauer J, *et al.* Sarcopenia: revised European consensus on definition and diagnosis. *Age Ageing* 2019; **48**: 16–31.

5 Wu Y, Wang W, Liu T, Zhang D. Association of Grip Strength With Risk of All-Cause Mortality, Cardiovascular Diseases, and Cancer in Community-Dwelling Populations: A Meta-analysis of Prospective Cohort Studies. *J Am Med Dir Assoc* 2017; **18**: 551.e17-551.e35.

6 Rijk JM, Roos PR, Deckx L, van den Akker M, Buntinx F. Prognostic value of handgrip strength in people aged 60 years and older: A systematic review and meta-analysis. *Geriatr Gerontol Int* 2015; published online Mai. DOI:10.1111/ggi.12508.

For peer review only - http://bmjopen.bmj.com/site/about/guidelines.xhtml

7 Mainous III AG, Tanner RJ, Anton SD, Jo A. Grip Strength as a Marker of Hypertension and Diabetes in Healthy Weight Adults. *Am J Prev Med* 2015. DOI:10.1016/j.amepre.2015.05.025.

8 Bhasin S, Travison TG, Manini TM, *et al.* Sarcopenia Definition: The Position Statements of the Sarcopenia Definition and Outcomes Consortium. *J Am Geriatr Soc* 2020; **68**: 1410–8.

9 Martin-Ruiz C, von Zglinicki T. Biomarkers of healthy ageing: expectations and validation. *Proc Nutr Soc* 2014; **73**: 422–9.

10 Leong DP, Teo KK, Rangarajan S, *et al.* Prognostic value of grip strength: findings from the Prospective Urban Rural Epidemiology (PURE) study. *The Lancet* 2015; **386**: 266–73.

11 Bohannon RW. Muscle strength: clinical and prognostic value of hand-grip dynamometry. *Curr Opin Clin Nutr Metab Care* 2015; **18**: 465–70.

12 Kuh D, Karunananthan S, Bergman H, Cooper R. A life-course approach to healthy ageing: maintaining physical capability. *Proc Nutr Soc* 2014; **73**: 237–48.

13 Lera L, Albala C, Leyton B, *et al.* Reference values of hand-grip dynamometry and the relationship between low strength and mortality in older Chileans. *Clin Interv Aging* 2018; **Volume 13**: 317–24.

14 Laukkanen JA, Voutilainen A, Kurl S, Araujo CGS, Jae SY, Kunutsor SK. Handgrip strength is inversely associated with fatal cardiovascular and all-cause mortality events. *Ann Med* 2020; **52**: 109–19.

15 Volaklis KA, Halle M, Meisinger C. Muscular strength as a strong predictor of mortality: A narrative review. *Eur J Intern Med* 2015; **26**: 303–10.

16 Zhuang C, Zhang F, Li W, *et al.* Associations of low handgrip strength with cancer mortality: a multicentre observational study. *J Cachexia Sarcopenia Muscle* 2020; **11**: 1476–86.

17 Xu X, Yang Z, Ma T, *et al.* The cut-off values of handgrip strength and lean mass index for sarcopenia among patients on peritoneal dialysis. *Nutr Metab* 2020; **17**: 84.

18 Alley DE, Shardell MD, Peters KW, *et al.* Grip strength cutpoints for the identification of clinically relevant weakness. *J Gerontol A Biol Sci Med Sci* 2014; **69**: 559–66.

19 McGrath R, Vincent BM, Peterson MD, *et al.* Weakness May Have a Causal Association With Early Mortality in Older Americans: A Matched Cohort Analysis. *J Am Med Dir Assoc* 2019; published online Nov 27. DOI:10.1016/j.jamda.2019.10.016.

20 Steiber N. Strong or Weak Handgrip? Normative Reference Values for the German Population across the Life Course Stratified by Sex, Age, and Body Height. *PLOS ONE* 2016; **11**: e0163917.

21 Kallman DA, Plato CC, Tobin JD. The role of muscle loss in the age-related decline of grip strength: cross-sectional and longitudinal perspectives. *J Gerontol* 1990; **45**: M82-88.

22 Dodds RM, Syddall HE, Cooper R, *et al.* Grip Strength across the Life Course: Normative Data from Twelve British Studies. *PLoS ONE* 2014; **9**: e113637.

23 Duchowny K, Peterson MD, Clarke P. Cut points for Clinical Muscle Weakness Among Older Americans. *Am J Prev Med* 2017; **53**: 63–9.

24 HRS. Health and Retirement Study, public use dataset. Produced and distributed by the University of Michigan with funding from the National Institute on Aging (grant number NIA U01AG009740). Ann Arbor, MI http://hrsonline.isr.umich.edu/. 2015.

For peer review only

For peer review only - http://bmjopen.bmj.com/site/about/guidelines.xhtml

25 Roberts HC, Denison HJ, Martin HJ, *et al.* A review of the measurement of grip strength in clinical and epidemiological studies: towards a standardised approach. *Age Ageing* 2011; **40**: 423–9.

26 Sanderson WC, Scherbov S. Measuring the Speed of Aging across Population Subgroups. *PLoS ONE* 2014; **9**: e96289.

27 Ortega FB, Silventoinen K, Tynelius P, Rasmussen F. Muscular strength in male adolescents and premature death: cohort study of one million participants. *The BMJ* 2012; **345**: e7279.

28 Cooper R, Kuh D, Hardy R, Mortality Review Group, on behalf of the FALCon and HALCyon study teams. Objectively measured physical capability levels and mortality: systematic review and meta-analysis. *BMJ* 2010; **341**: c4467–c4467.

29 Duchowny K. Do Nationally Representative Cutpoints for Clinical Muscle Weakness Predict Mortality? Results From 9 Years of Follow-up in the Health and Retirement Study. *J Gerontol Ser A* 2019; **74**: 1070–5.

30 Bachettini NP, Bielemann RM, Barbosa-Silva TG, Menezes AMB, Tomasi E, Gonzalez MC. Sarcopenia as a mortality predictor in community-dwelling older adults: a comparison of the diagnostic criteria of the European Working Group on Sarcopenia in Older People. *Eur J Clin Nutr* 2020; **74**: 573–80.

For peer review only

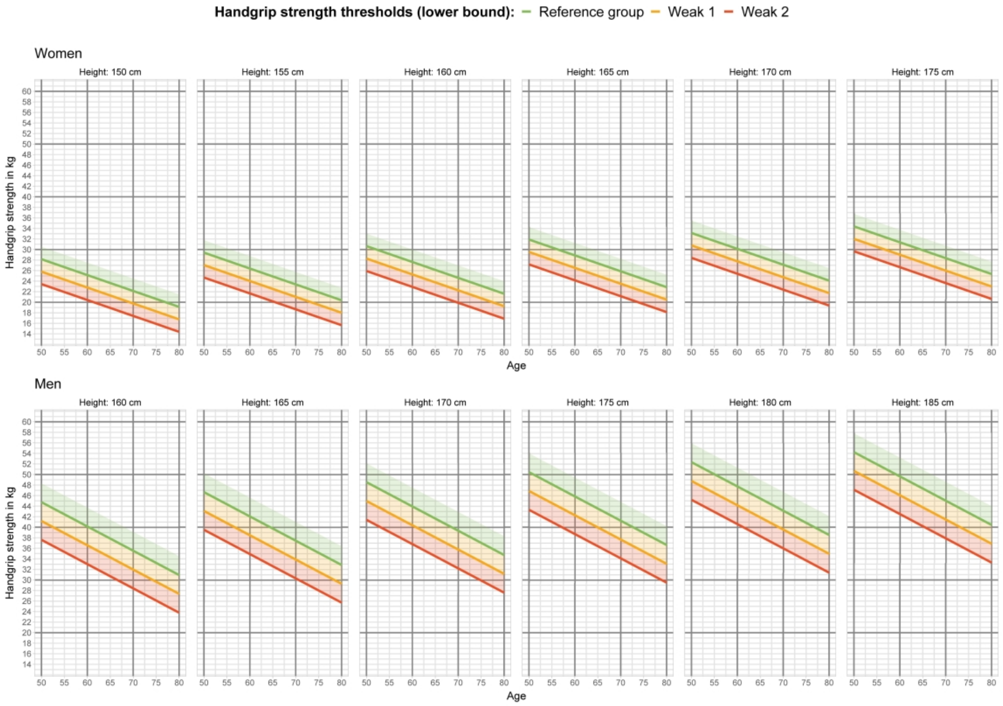

Figure 1: Model-based HGS thresholds (lower bound) in kg by gender, age, and body height. The reference group refers to HGS at or slightly above the mean [0.0 SD, 0.5 SD), Weak 1 to HGS up to 0.5 SD below the mean [-0.5 SD, 0.0 SD), and Weak 2 to less than a half to a full SD below the mean [-1.0 SD, -0.5 SD).

549x399mm (300 x 300 DPI)

SUPPLEMENTARY MATERIAL

Initial sample

N = 12,137 (6,931 women; 5,206 men)

↓

Sample after considering demographic characteristics, anthropometric traits, and HGS

Keeping individuals aged 50 to 80;
keeping Caucasian individuals;
excluding male outliers with height < 150 cm or height > 210 cm;
excluding female outliers with height < 130 cm or height > 190 cm;
excluding outliers with weight < 30 kg or weight > 150 kg;
excluding outliers with handgrip strength < 6 kg or handgrip strength > 80 kg

N = 8,309 (4,668 women; 3,641 men)

↓

Sample after dropping illogical exposure

Excluding individuals that supposedly died before the survey took place

N = 8,306 (4,666 women; 3,640 men)

↓

Final sample after excluding observations with residuals larger than 2.5 SD

N = 8,156 (4,573 women; 3,583 men)

Figure A1: Sample construction

For peer review only

For peer review only - http://bmjopen.bmj.com/site/about/guidelines.xhtml

	Model 1 (without education)	Model 2 (with education)
**Men**		
Reference: [0.0 SD, 0.5 SD)	1.00 (reference)	1.00 (reference)
Strong: [0.5 SD, 3.0 SD)	0.93 (0.69-1.25)	0.93 (0.69-1.26)
Weak 1: [-0.5 SD, 0.0 SD)	1.67 (1.23-2.26)	1.68 (1.24-2.27)
Weak 2: [-1.0 SD, -0.5 SD)	2.02 (1.49-2.75)	1.98 (1.46-2.69)
Weak 3: [-2.0 SD, -1.0 SD)	2.40 (1.77-3.26)	2.39 (1.76-3.25)
Weak 4: [-3.0 SD, -2.0 SD)	2.34 (1.40-3.93)	2.32 (1.39-3.89)
Age in years	1.09 (1.07-1.10)	1.09 (1.07-1.10)
Years of education		0.96 (0.93-0.98)
Max log likelihood	-2358.9	-2353.0
LR test statistic	244.6	256.4
Total time at risk	21864	21864
Events	529	529
**Women**		
Reference: [0.0 SD, 0.5 SD)	1.00 (reference)	1.00 (reference)
Strong: [0.5 SD, 3.0 SD)	0.90 (0.66-1.22)	0.89 (0.66-1.22)
Weak 1: [-0.5 SD, 0.0 SD)	1.32 (0.96-1.82)	1.30 (0.94-1.79)
Weak 2: [-1.0 SD, -0.5 SD)	1.65 (1.20-2.28)	1.62 (1.18-2.24)
Weak 3: [-2.0 SD, -1.0 SD)	1.85 (1.34-2.55)	1.83 (1.32-2.53)
Weak 4: [-3.0 SD, -2.0 SD)	3.03 (1.83-5.04)	2.98 (1.79-4.96)
Age in years	1.10 (1.08-1.12)	1.10 (1.08-1.11)
Years of education		0.93 (0.90-0.96)
Max log likelihood	-2192.8	-2181.7
LR test statistic	212.6	234.8
Total time at risk	28500	28500
Events	449	449

95% CI in parentheses, years of education < 20 years

Table A1: Hazard ratios for mortality by gender and st_HGS group, with and without years of education as control

For peer review only

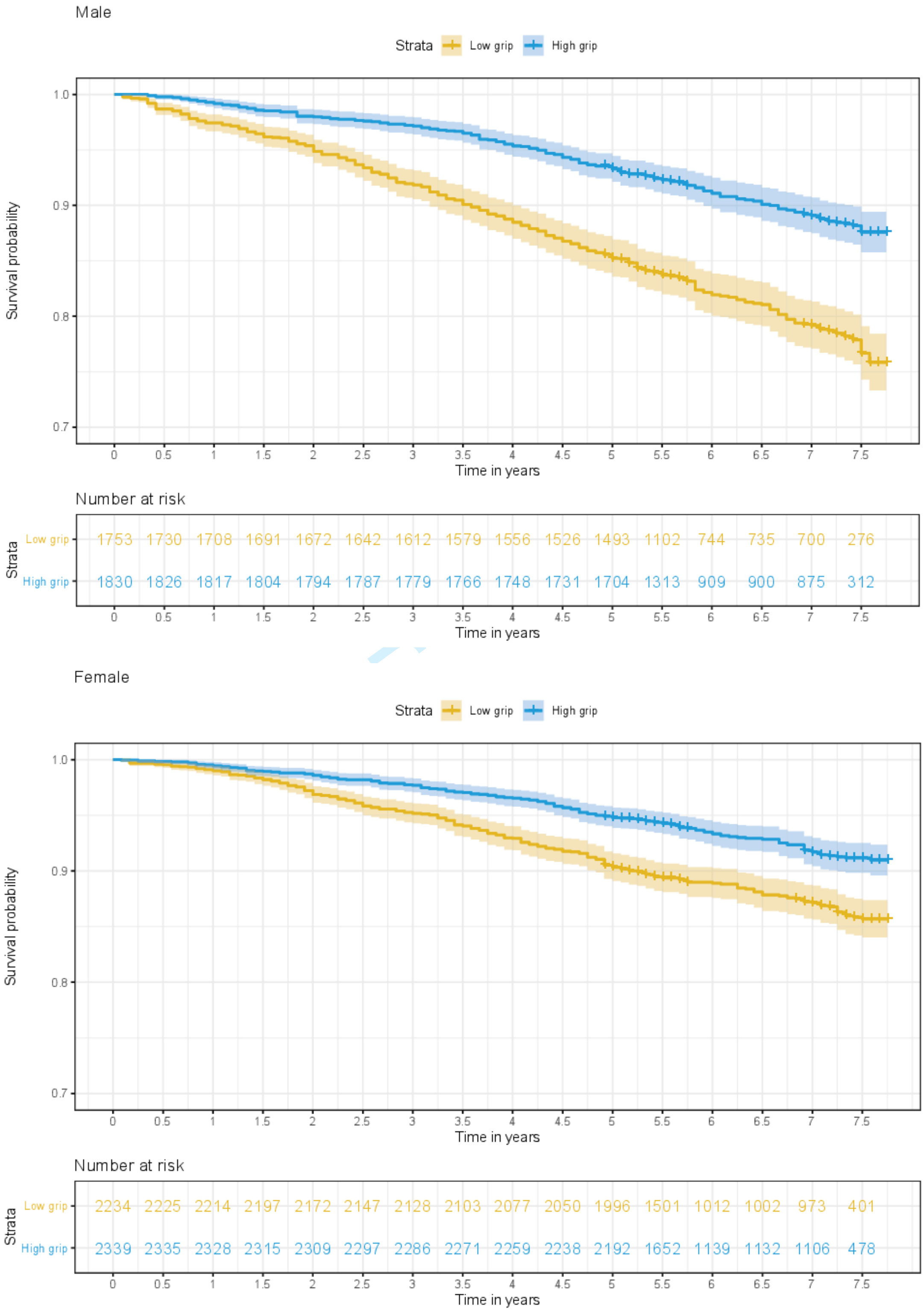

Figure A2: Survival and numbers at risk, by gender and standardised handgrip strength

“High grip” refers to the strong and reference group and “low grip” to groups Weak1-4

STROBE Statement—Checklist of items that should be included in reports of ***cross-sectional studies***

	Item No	Recommendation	Page No
**Title and abstract**	1	(*a*) Indicate the study's design with a commonly used term in the title or the abstract	2
		(*b*) Provide in the abstract an informative and balanced summary of what was done and what was found	2
**Introduction**			
Background/rationale	2	Explain the scientific background and rationale for the investigation being reported	2-5
Objectives	3	State specific objectives, including any prespecified hypotheses	2,5
**Methods**			
Study design	4	Present key elements of study design early in the paper	2,5
Setting	5	Describe the setting, locations, and relevant dates, including periods of recruitment, exposure, follow-up, and data collection	5-6
Participants	6	(*a*) Give the eligibility criteria, and the sources and methods of selection of participants	5-6
Variables	7	Clearly define all outcomes, exposures, predictors, potential confounders, and effect modifiers. Give diagnostic criteria, if applicable	5-8
Data sources/ measurement	8*	For each variable of interest, give sources of data and details of methods of assessment (measurement). Describe comparability of assessment methods if there is more than one group	*5-6*
Bias	9	Describe any efforts to address potential sources of bias	8
Study size	10	Explain how the study size was arrived at	5-6
Quantitative variables	11	Explain how quantitative variables were handled in the analyses. If applicable, describe which groupings were chosen and why	5-8
Statistical methods	12	(*a*) Describe all statistical methods, including those used to control for confounding	5-8
		(*b*) Describe any methods used to examine subgroups and interactions	5-8
		(*c*) Explain how missing data were addressed	5-8
		(*d*) If applicable, describe analytical methods taking account of sampling strategy	5-8
		(*e*) Describe any sensitivity analyses	5-8
**Results**			
Participants	13*	(a) Report numbers of individuals at each stage of study—eg numbers potentially eligible, examined for eligibility, confirmed eligible, included in the study, completing follow-up, and analysed	5-10
		(b) Give reasons for non-participation at each stage	5-10
		(c) Consider use of a flow diagram	NA
Descriptive data	14*	(a) Give characteristics of study participants (eg demographic, clinical, social) and information on exposures and potential confounders	5-10
		(b) Indicate number of participants with missing data for each variable of interest	NA
Outcome data	15*	Report numbers of outcome events or summary measures	5-10
Main results	16	(*a*) Give unadjusted estimates and, if applicable, confounder-adjusted estimates and their precision (eg, 95% confidence interval). Make clear which confounders were adjusted for and why they were included	2-13

For peer review only - http://bmjopen.bmj.com/site/about/guidelines.xhtml

		(*b*) Report category boundaries when continuous variables were categorized	NA
		(*c*) If relevant, consider translating estimates of relative risk into absolute risk for a meaningful time period	NA
Other analyses	17	Report other analyses done—eg analyses of subgroups and interactions, and sensitivity analyses	5-8
**Discussion**			
Key results	18	Summarise key results with reference to study objectives	8-13
Limitations	19	Discuss limitations of the study, taking into account sources of potential bias or imprecision. Discuss both direction and magnitude of any potential bias	12-13
Interpretation	20	Give a cautious overall interpretation of results considering objectives, limitations, multiplicity of analyses, results from similar studies, and other relevant evidence	12-13
Generalisability	21	Discuss the generalisability (external validity) of the study results	12-13
**Other information**			
Funding	22	Give the source of funding and the role of the funders for the present study and, if applicable, for the original study on which the present article is based	13

*Give information separately for exposed and unexposed groups.

Note: An Explanation and Elaboration article discusses each checklist item and gives methodological background and published examples of transparent reporting. The STROBE checklist is best used in conjunction with this article (freely available on the Web sites of PLoS Medicine at http://www.plosmedicine.org/, Annals of Internal Medicine at http://www.annals.org/, and Epidemiology at http://www.epidem.com/). Information on the STROBE Initiative is available at www.strobe-statement.org.

For peer review only

For peer review only - http://bmjopen.bmj.com/site/about/guidelines.xhtml
